# Multiresonant Nondispersive Infrared Gas Sensing: Breaking the Selectivity and Sensitivity Trade-Off

**DOI:** 10.1021/acsphotonics.5c02787

**Published:** 2026-03-12

**Authors:** Emma R. Bartelsen, J. Ryan Nolen, Christopher R. Gubbin, Mingze He, Ryan W. Spangler, Joshua Nordlander, Cassandra L. Bogh, Katja Diaz-Granados, Simone De Liberato, Jon-Paul Maria, James R. McBride, Joshua D. Caldwell

**Affiliations:** † Interdisciplinary Materials Science Program, 5718 Vanderbilt University, Nashville 37240, Tennessee, United States; ‡ Department of Mechanical Engineering, Vanderbilt University, Nashville 37235, Tennessee, United States; § Sensorium Technological Laboratories, 6714 Duquaine Ct, Nashville 37205, Tennessee, United States; ∥ Photonics Initiative, Advanced Science Research Center, City University of New York, New York 10031, New York, United States; ⊥ Department of Materials Science and Engineering, 311285 The Pennsylvania State University, University Park 16802, Pennsylvania, United States; # Istituto di Fotonica e Nanotecnologie, Consiglio Nazionale delle Ricerche (CNR), Piazza Leonardo da Vinci 32, Milano 20133, Italy; ¶ School of Physics and Astronomy, 7423 University of Southampton, University Road, Southampton SO17 1BJ, U.K.; ∇ Department of Chemistry, The Vanderbilt Institute of Nanoscale Science and Engineering, Vanderbilt University, Nashville 37235, Tennessee, United States

**Keywords:** multiresonant thermal emitters, gas sensing, filterless infrared sensing, aperiodic distributed Bragg reflectors (a-DBRs), Tamm plasmon polaritons

## Abstract

In applications such as atmospheric monitoring of greenhouse gases and pollutants, the detection and identification of trace concentrations of harmful gases is commonly achieved using nondispersive infrared (NDIR) sensors. These devices typically employ a broadband infrared emitter, thermopile detector, and spectrally selective bandpass filter tuned to the vibrational resonance of the target analyte. However, fabrication of these filters is costly and limited to a single frequency. This limitation introduces a fundamental trade-off, as broadening the optical passband width enhances sensitivity but compromises selectivity, whereas narrowing improves selectivity at the expense of sensitivity. In this work, we validate a filterless NDIR gas sensing approach utilizing a multipeak thermal emitter developed through an inverse design. This emitter enhances detection sensitivity by simultaneously targeting multiple absorption bands, demonstrated through the creation of a sensor designed for the C–H vibrational modes of propane (C_3_H_8_). Additionally, a second set of single-peak emitters was developed to showcase the capability of designing highly selective sensors operating within close spectral proximity. These emitters, targeting the stretching modes of carbon monoxide (CO) and carbon dioxide (CO_2_), exhibit quality factors (Q-factors) above 50 and minimal crosstalk, enabling accurate detection of the target gas without interference from gases with spectrally adjacent absorption bands. This is enabled by aperiodic distributed Bragg reflectors (a-DBRs), which achieve higher Q-factors with fewer layers than periodic Bragg reflectors. Experimental results demonstrate that this approach breaks the trade-off between sensitivity and selectivity.

## Graphical abstract

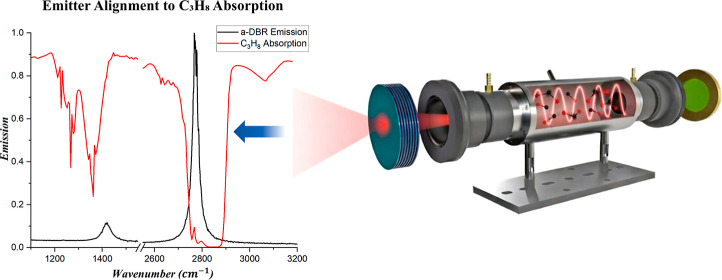

## Introduction

The characteristic, infrared-active, vibrational resonances of a molecule populate the mid-infrared (MIR) spectral range, forming a unique spectral “fingerprint”. This “fingerprint” provides insights about the molecular structure and composition.^1^ Optical techniques are commonly employed to exploit this MIR “fingerprint” to determine the presence and concentration of gas-phase chemical species in industrial,^2^ medical,^3–5^ defense,^6,7^ and research settings.^8^ Some applications include atmospheric sensing of greenhouse gases^6,9^ and other pollutants^10^ and monitoring of manufacturing gases^11^ and leak detection for workplace safety. While spectroscopic techniques such as Fourier transform infrared (FTIR) spectroscopy are widely used in laboratory settings and some industrial environments, their large footprint and high cost, together with the interferometer’s sensitivity to vibrations, can limit practicality in compact, field-deployable sensing systems. Furthermore, these techniques often require postprocessing to extract specific information such as gas classification and concentration, making them less suitable for real-time, application-specific sensing.^12,13^

To address the size and cost limitations of traditional spectroscopic techniques, many commercial applications employ nondispersive infrared (NDIR) gas sensors.^2^ These are compact optical devices that exploit the Beer–Lambert law^14,15^ [eq 1]:

1 A=εbC

where *A* is the absorbance, ε is the molar absorptivity, *b* is the path length, and *C* is the concentration. Thus, the concentration of a gas can be determined via a reduction in transmitted light, where absorption at resonant vibrational frequencies over a defined path length and cross-sectional area is directly proportional to the concentration of that molecule.

Traditional NDIR systems typically incorporate a broadband infrared light source, a thermopile or pyroelectric detector, and a spectrally selective bandpass filter tuned to one of the strongest vibrational resonances for the target gas, within a gas cell of a defined optical path length. One of the main limitations of NDIR systems is that these filters are limited to a single spectral band. While a filter with a sufficiently narrow passband enables single-gas selectivity, it compromises overall sensitivity by blocking a large fraction of the thermally emitted radiation, often including some of the absorption bands of the molecule of interest. Systems capable of detecting multiple gas types often incorporate a rotating filter wheel with multiple bandpass filters, which increases the size, complexity, and cost of the sensor. A potential solution to mitigate the limitations of single-frequency bandpass filters is to integrate narrowband detectors into the system. Such narrowband detectors have been demonstrated using materials such as graphene resonators or plasmonic structures.^16,17^ However, they often require nanoscale lithographic fabrication, which increases the cost and limits scalability. Another alternative is the use of narrowband sources, such as quantum cascade lasers (QCLs) that offer high performance but come with high power demands and are not cost-effective for widespread industrial use.^18^ Mid-infrared LEDs have gained interest due to their compact form, but they suffer from low output power and broadband emission profiles, which may potentially result in spectral crosstalk between different gas types, resulting in false positives.^19,20^ Recent advancements in nanophotonics have enabled the development of narrowband infrared sources using photonic crystals,^21–23^ structured polaritonic materials,^24–29^ and metamaterial-based designs.^30–32^ These nanophotonic infrared emitting metamaterials (NIREMs) can exhibit line widths approaching those of molecular vibrational features, enabling more sensitive and selective gas detection.^26,29,33,34^ However, these systems still rely on complex fabrication processes, such as electron beam lithography, to pattern polaritonic nanostructures and stimulate polariton resonances in the MIR.

Tamm plasmon polaritons (TPPs) are formed at the interface between a distributed Bragg reflector (DBR) and a plasmonic medium, supporting omnidirectional, spectrally narrow resonances that can be engineered for selective MIR emission. These modes arise from the interplay between the high reflectivity of the Bragg stack and the metallic response of the plasmonic layer, which together establishes the phase-matching conditions necessary for an interface-bound electromagnetic mode. Within the DBR, multiple reflections at the dielectric interfaces lead to frequency-dependent constructive and destructive interference, producing a photonic bandgap that confines the electromagnetic field near the interface. In contrast to typical photonic bandgaps, the evanescent nature of the thermal emission from the metallic component results in a passband for these fields. Thus, when these interference conditions are satisfied, the resulting TPP mode is localized at the plasmonic boundary while extending evanescently into the Bragg stack, yielding spectrally narrow resonances at the frequencies of the defined photonic bandgaps.

Early experiments by Yang et al. showed narrowband, wavelength-selective thermal emission from Tamm structures, establishing their potential as compact, lithography-free emitters for sensing.^35^ Building on these findings, our recent work demonstrated deterministic inverse design of Tamm-based thermal emitters with single and multiresonant control using aperiodic Bragg stacks on tunable cadmium oxide (CdO), making them well suited for gas sensing applications.^36^ We further showed that coupling Tamm plasmon and Tamm phonon polaritons yields high-Q, multiband planar absorbers with fewer Bragg layers, simplifying fabrication while preserving narrow line widths.^34^ Together, these studies indicated the promise of enhanced, filterless NDIR sensing, but this has not yet been validated, motivating the approach detailed below.

In this work, we present a lithography-free, multifrequency NDIR approach that relies on planar frequency-selective emitters supported by dielectric stacks. Using the stochastic gradient descent inverse design algorithm referenced above,^36^ we demonstrate control over thermal emission profiles tailored to spectral targets of varying complexity. While prior implementations of this inverse-design approach focused on TPP-supporting emitter configurations, the underlying interference-based photonic mechanisms are not restricted to TPP formation alone. Here, we design and experimentally validate both TPP-supporting emitters (CO and CO_2_) and an emitter that does not support TPPs (C_3_H_8_). In the non-TPP-supporting structure, spectrally selective thermal emission is achieved through frequency-dependent constructive and destructive interference within the dielectric Bragg stack, enabling narrowband emission without a plasmonic interface. By validating both TPP-supporting and non-TPP-supporting emitters within a tabletop, filterless NDIR gas-cell configuration, this work demonstrates gas sensing using inversely designed thermal emitters.

These filterless NDIR schemes improve efficiency by eliminating off-resonant emission losses and reduce design complexity, supporting compact integration and the potential for multi-gas sensing on a single platform. Our design overcomes the limitations of conventional NDIR systems by eliminating the need for bandpass filters, narrowband detectors, or lithographically patterned metasurfaces while preserving a compact form factor, cost efficiency, planar emitter design, and the sensitivity required for real-world gas sensing applications.

## Results

For each target gas, the desired emission spectra were defined based on the MIR vibrational absorption features of the analyte, obtained from established databases such as NIST.^37^ Target emission wavelengths were chosen to align with the target molecular absorption bands while minimizing overlap with spectrally closely interfering species. The emission line width and intensity were then tailored to match the width and amplitude of the absorption features over the expected concentration range of the gas.^36^

The emission wavelength, amplitude, and line width of periodic DBR-based emitters are determined by four parameters: the thickness of the constituent layers within a period, the number of periods, the materials chosen for the high- and low-refractive index layers, and the optical properties of the adjacent reflective film.^38^ Achieving a spectrally narrow emission with periodic stacks often requires a large number of layers to strengthen confinement and narrow the line width. This, in turn, complicates fabrication by demanding high precision in each layer, increasing cost and raising the probability of interfacial defects from the many material interfaces. Furthermore, periodic stacks cannot generate arbitrary multipeak emissions but only a single resonance and its harmonics. To achieve independent control over multiple peaks, periodicity must be broken. Breaking the periodic structure allows each layer thickness to be tuned independently, yielding aperiodic DBRs (a-DBRs) that provide far greater design flexibility, and can realize complex spectral responses with fewer layers than periodic stacks.^34,36^ However, the expanded design space introduces substantial complexity, making manual forward, intuition-based design approaches infeasible and necessitating the use of inverse design or machine learning techniques to efficiently identify structures that meet the desired spectral targets.

To address the expanded parameter space, we utilized our group’s stochastic gradient descent inverse design algorithm to realize emitters with tailored, narrowband thermal emission profiles.^36^ This approach provides precise control over emission wavelengths and line widths, enabling simultaneous targeting of multiple molecular vibrational modes. Such multiresonant profiles improve sensitivity without compromising selectivity in a filterless NDIR configuration. To validate this approach, we performed two experiments. First, we designed a dual-peak emitter targeting the C–H deformation (1300–1500 cm^–1^) and stretching (2800–3000 cm^–1^) modes of propane (C_3_H_8_),^39^ enabling filterless NDIR sensing of both resonances in a single measurement, as illustrated in Figure 1b. Second, we designed single-peak emitters for carbon monoxide (CO) and carbon dioxide (CO_2_), targeting the stretching mode at 2150 cm^–1^ and the asymmetric stretching mode at 2349 cm^–1^, respectively, to test spectral selectivity for closely spaced absorption features.^40,41^ Both emitters exhibited Q-factors above 50, effectively isolating their target resonances and minimizing spectral crosstalk. Combined, we quantify and validate the enhancement in NDIR sensitivity through multimode targeting, while maintaining high selectivity, as confirmed by the lack of detection of nontarget gases with absorption bands in close spectral proximity.

**Figure 1:**
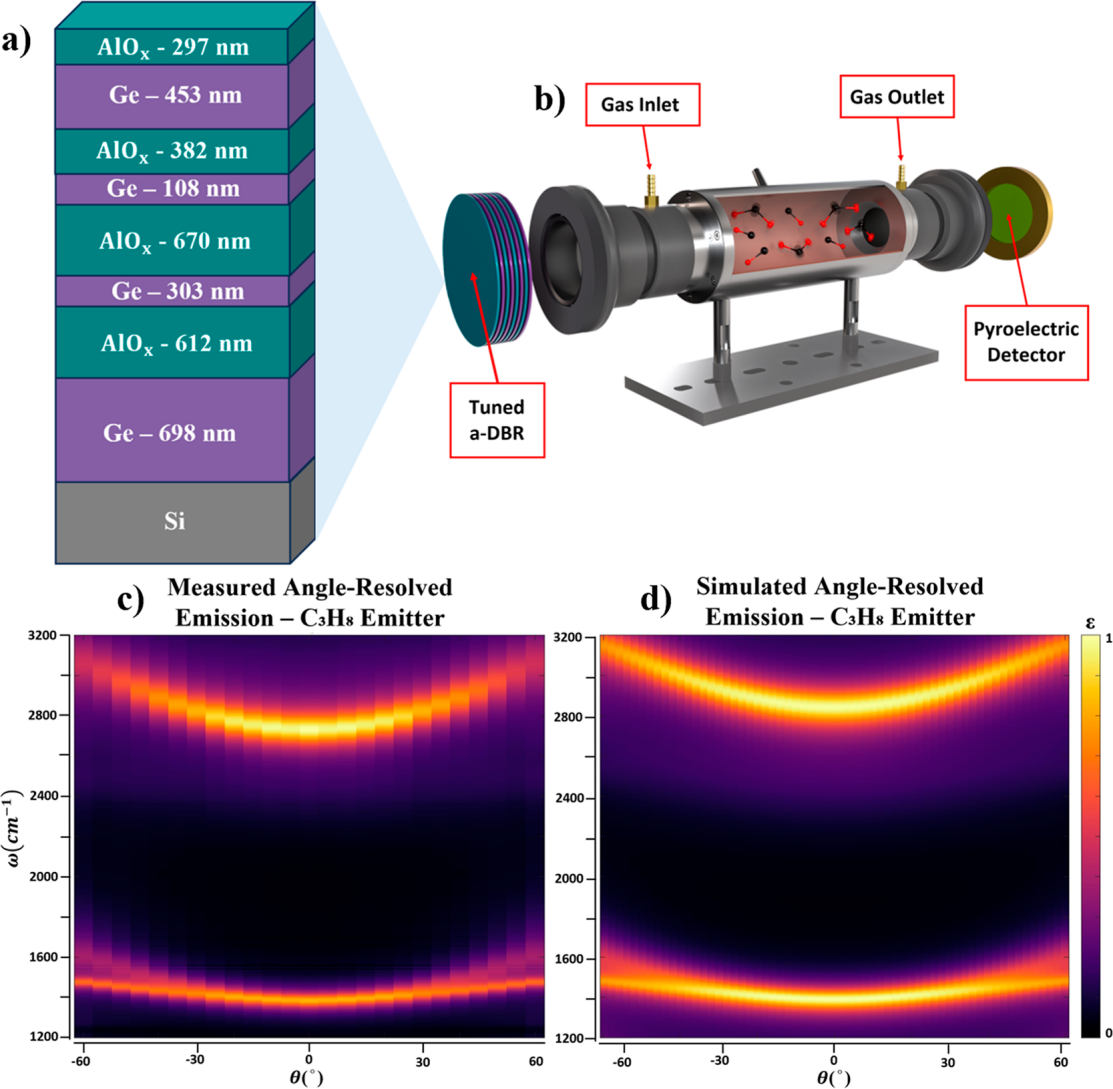
(a) C_3_H_8_ emitter proposed design. (b) Filterless NDIR gas sensing setup, targeting multiple vibrational resonances simultaneously. (c) Angle-resolved emission measurements from the C_3_H_8_ emitter. (d) Angle-resolved emission simulation results for the proposed C_3_H_8_ emitter.

### C_3_H_8_ Emitter

To enable multiresonant emission for C_3_H_8_ detection, we fabricated a thermal emitter comprising an a-DBR stack of eight alternating Ge and AlO_*x*_ layers on a low-resistivity Si substrate (layer design shown in [Figure 1a]). Target and as-grown layer thicknesses are listed in the Supporting Information. The a-DBR was designed to produce thermal emission at two distinct peaks, with the lower-frequency band centered at 1420 cm^–1^ (C–H deformation) and a higher-frequency band centered at 2768 cm^–1^ (C–H stretching) [Figure 1c]. These peaks align closely with the target and calculated spectra of [Figure 1d], demonstrating the strong spectral tunability of this approach and its potential for engineering emitters with precisely tailored spectral profiles.

Along the surface normal, the emitter exhibited a strong overlap with the C–H deformation mode of C_3_H_8_, with emissivity ε ≈ 0.98 at 1420 cm^–1^ (fwhm = 61.15 cm^–1^). The higher-frequency band peaked at 2768 cm^–1^ (ε ≈ 0.98, fwhm = 29.5 cm^–1^), slightly red-shifted from the C–H stretching mode. Both peaks blue-shifted with angle, with the lower-frequency band showing a small curvature (*b_l_* = 0.0278 $\frac{cm^{-1}}{deg^{2}}$ ) that maintained a strong overlap with the C_3_H_8_ absorption feature up to 60°. The higher-frequency band exhibited a larger curvature (*b_u_* = 0.2583 $\frac{cm^{-1}}{deg^{2}}$ ), maintaining overlap up to 33° [Figure 1c]. These angle-resolved measurements were acquired using a custom-built FTIR-based heated rotation stage, described in greater detail in the Methods section. The measured angle-resolved emissivity spectra at 300 °C showed excellent agreement with transfer-matrix-based absorptivity calculations [Figure 1d].^42^ Details regarding the thermal emission measurements, including the schematic of the experimental setup, can be found in the Supporting Information.

To assess the thermal stability of the emitter, we measured its spectral output from 200 to 300 °C using our FTIR system [Figure 2a]. The emission spectra show minimal spectral shifting across this temperature range, with peak positions remaining well-aligned with the vibrational absorption bands of C_3_H_8_. This consistency is critical for reliable sensing, as it ensures that the emitted wavelengths remain within the absorption features of the target analyte.

**Figure 2:**
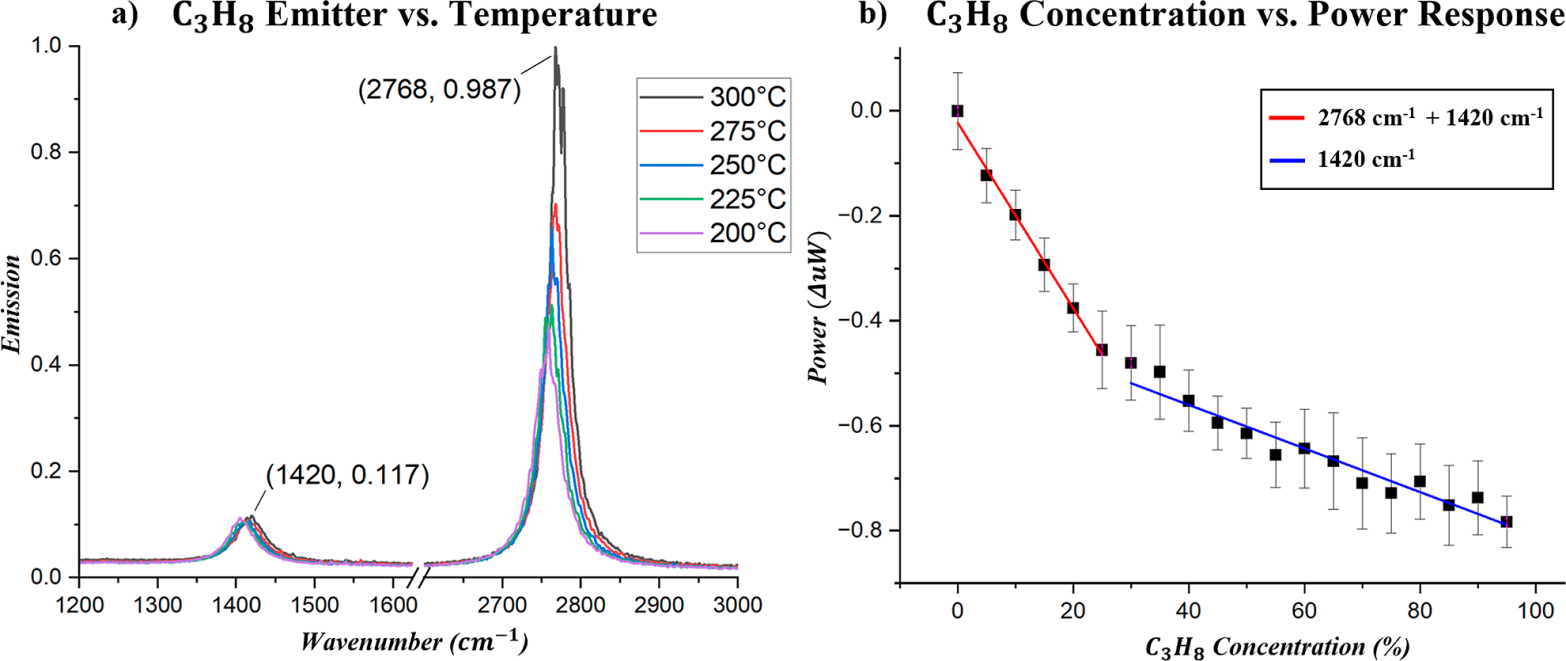
(a) Emission spectra of C_3_H_8_ emitter measured from 200 to 300 °C, showing minimal spectral shift and consistent alignment with the target absorption bands across the temperature range. (b) Measured power within custom-built NDIR gas sensing setup, depicting decreasing signal with increasing C_3_H_8_ concentration and enhanced sensitivity at concentrations below 10% volume.

We then evaluated the dual-peak emitter in a custom NDIR gas sensing setup (schematic in the Supporting Information). The emitter was operated at 300 °C, and off-normal emission was collected with a Winston cone to maximize the overlap with the C_3_H_8_ absorption features. Propane concentrations were varied from 0 to 400,000 ppm (0–40% by volume). As the concentration increased, the detected power decreased in accordance with the Beer–Lambert behavior [Figure 2b]. The dual-band design enhanced sensitivity in the 0–100,000 ppm range (0–10%), yielding a slope approximately 4.25× steeper than at higher concentrations. At concentrations above 10%, the 2768 cm^–1^ band saturated, leaving the response dominated by the lower-frequency mode. These results highlight the advantage of the dual-band design, which improves sensitivity in the 0–10% range compared to conventional single-band NDIR systems while maintaining a wide dynamic range. Simulations confirm strong overlap between the emitter output and the vibrational resonances of C_3_H_8_ at 1420 cm^–1^ and 2768 cm^–1^, with both bands contributing to absorbance and producing a larger power drop than either band alone (Supporting Information).

To evaluate performance relative to commercial systems, the propane emitter was compared to a single-band InfraTec NDIR filter centered at 2678 cm^–1^ with a fwhm of 63.5 cm^–1^.^43^ The filter’s broader 63.5 cm^–1^ fwhm, compared to our emitter’s 29.5 cm^–1^ line width at 2768 cm^–1^, yielded slightly higher sensitivity, with the emitter exhibiting an 8% decrease in sensitivity for this single resonance. When accounting for both resonant modes of the emitter (2768 cm^–1^ and 1420 cm^–1^), the dual-band design exhibited a 36.5% increase in sensitivity compared with the commercial filter. When the InfraTec filter fwhm was matched to that of the emitter (29.5 cm^–1^), the emitter exhibited a 92% increase in sensitivity for the 2768 cm^–1^ band alone and an 184% increase for the dual-band design. These results emphasize the trade-off between sensitivity and selectivity in traditional NDIR sensing and demonstrate that our a-DBR-based emitter design overcomes this limitation, enabling enhanced sensitivity without compromising spectral precision.

### CO and CO_2_ Emitters

In the previous section, we demonstrated that targeting multiple vibrational resonances can significantly enhance the sensitivity in a filterless NDIR setup. Traditional NDIR systems often face a trade-off between sensitivity and selectivity. Our approach overcomes this limitation by designing emitters with tailored spectral responses that achieve both. In this section, we focus on enhancing selectivity by using the same gradient descent optimization to design two high-Q emitters that independently target the stretching mode of CO (ω_CO_ = 2150 cm^–1^) and asymmetric stretching mode of CO_2_ (ω_CO_2__ = 2349 cm^–1^), respectively.^36^ These vibrational modes are spectrally adjacent but nonoverlapping, allowing high-Q emitters to selectively detect one gas without interference or false positives from the other.

The a-DBR was deposited on highly doped CdO (*N*_d_ = 3.85 × 10^20^ cm^–3^), whose metallic behavior in the MIR, resulting from both its negative real permittivity and non-negligible imaginary contribution, provides the near-unity reflectivity required for spectrally selective thermal emission with minimal optical losses.^44,45^ The high carrier density of CdO further allows tuning of its permittivity, offering additional flexibility in designing emission wavelengths.^44,46–50^ Each CO and CO_2_ wavelength-selective emitter consists of three alternating Ge and DyF_3_ layers in the a-DBR [Figure 3c,f)], providing the narrow line widths needed for selective gas detection.

**Figure 3:**
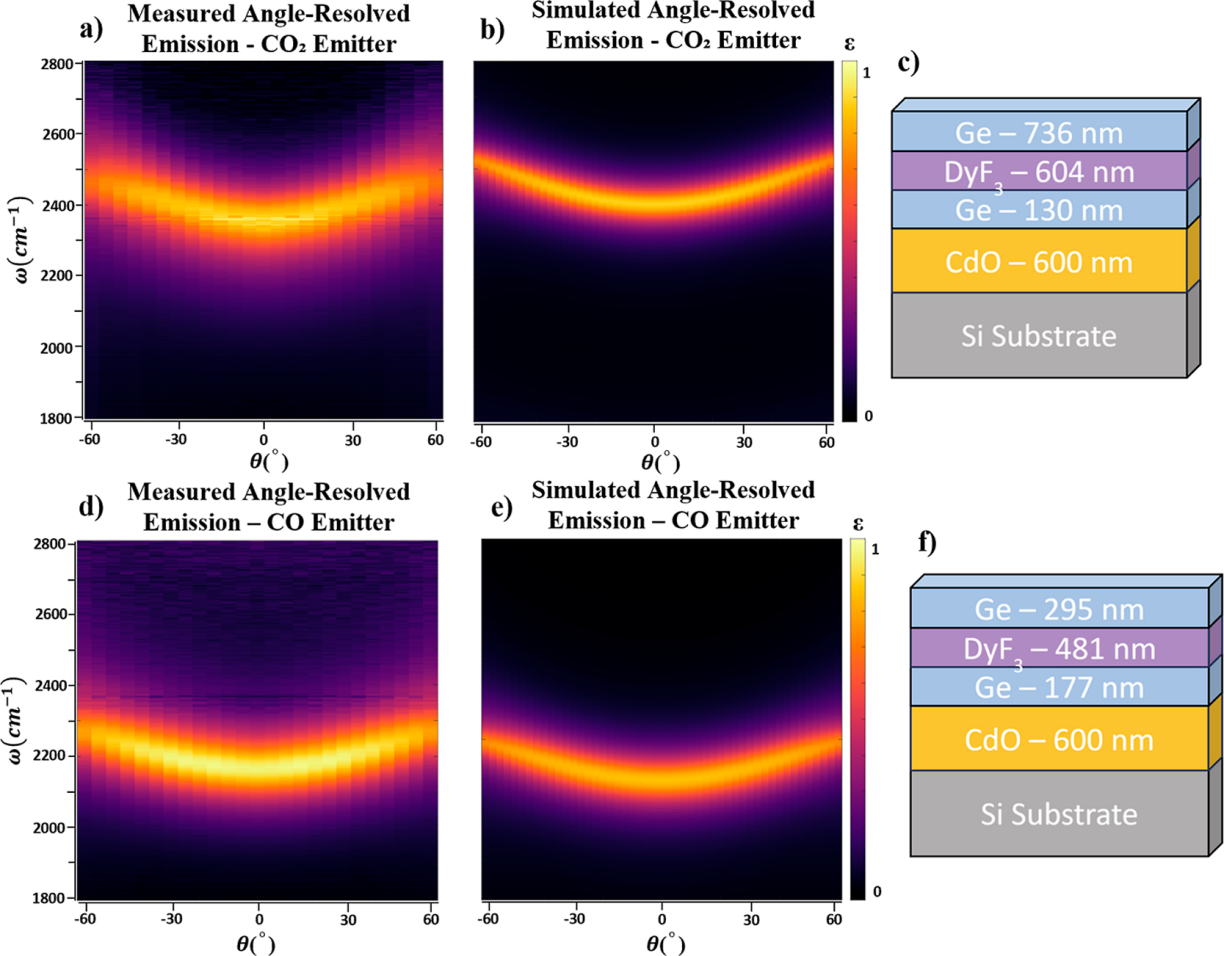
(a) Measured angle-resolved emission of CO_2_ emitter. (b) Simulated angle-resolved emission of CO_2_ emitter. (c) CO_2_ emitter structure with 3-layer a-DBR on doped CdO. (d) Measured angle-resolved emission of CO emitter. (e) Simulated angle-resolved emission of CO emitter. (f) CO emitter structure with 3-layer a-DBR on doped CdO.

Angle-resolved thermal emission measurements, performed using the previously described FTIR-based heated rotation stage, confirmed strong, narrow-band emission centered at the target frequencies for both emitters. The CO_2_-targeting emitter peaked at 2351 cm^–1^ with ε ≈ 0.98 and a Q-factor of 52.55 exhibiting high emissivity across the full CO_2_ absorption band [Figure 3a]. In comparison, the CO-targeting emitter peaked at 2146 cm^–1^ with ε ≈ 0.98 and a Q-factor of 76.7, similarly maintaining a strong overlap with the CO absorption band [Figure 3d]. To validate these findings, we also simulated the angular dispersion of both emitters. The CO_2_ emitter exhibited low angular dispersion (*b*_CO_2__ = 0.0806 $\frac{cm^{-1}}{deg^{2}}$ ), maintaining spectral alignment with the CO_2_ absorption band up to a collection angle of 57° [Figure 3b]. The CO emitter showed a similarly low angular dispersion (*b*_CO_ = 0.0594 $\frac{cm^{-1}}{deg^{2}}$ ), preserving overlap with the CO absorption band out to 60° [Figure 3e].

Similar to the C_3_H_8_ emitter, the thermal stability of the CO and CO_2_ emitters was also evaluated by measuring their spectral output from 200 to 300 °C using our FTIR system. Both emitters demonstrated minimal spectral shifting across this temperature range, with peak positions changing by only 0.23 cm^–1^/°C for CO and 0.25 cm^–1^/°C for CO_2_, remaining well-aligned with their respective absorption bands (Supporting Information). These results confirm that the CO and CO_2_ emitters maintain reliable performance under varying thermal conditions, further supporting their use in practical sensing environments.

Each emitter was again incorporated into our NDIR gas sensing setup (Supporting Information). For CO_2_ detection, the emitter was heated to 300 °C, and concentrations from 0 to 1000 ppm of CO_2_ were introduced into the gas cell. As expected, the detected power dropped in accordance with the Beer–Lambert behavior [Figure 4a]. To evaluate sensor selectivity, CO gas was flowed through the cell while the CO_2_ emitter remained active. Despite the close spectral proximity between the CO and CO_2_ absorption bands, the pyroelectric detector registered no measurable decrease in power [Figure 4b]. This confirms that the emitter’s output exhibits minimal overlap with CO absorption features, thereby avoiding false positives and demonstrating strong spectral selectivity critical for accurate gas identification. This procedure was then repeated with the CO emitter. As the CO concentrations increased, the detected power decreased [Figure 4c], confirming selective emission and absorption. However, a slight downward trend in detected power was observed as the CO_2_ concentration increased [Figure 4d]. This suggests a minor spectral overlap between the CO emitter output and the CO_2_ absorption features. This overlap of emissivity with other gas types can be mitigated by reducing the emission line width through the use of lower-loss dielectric materials or by incorporating additional a-DBR layers.

**Figure 4:**
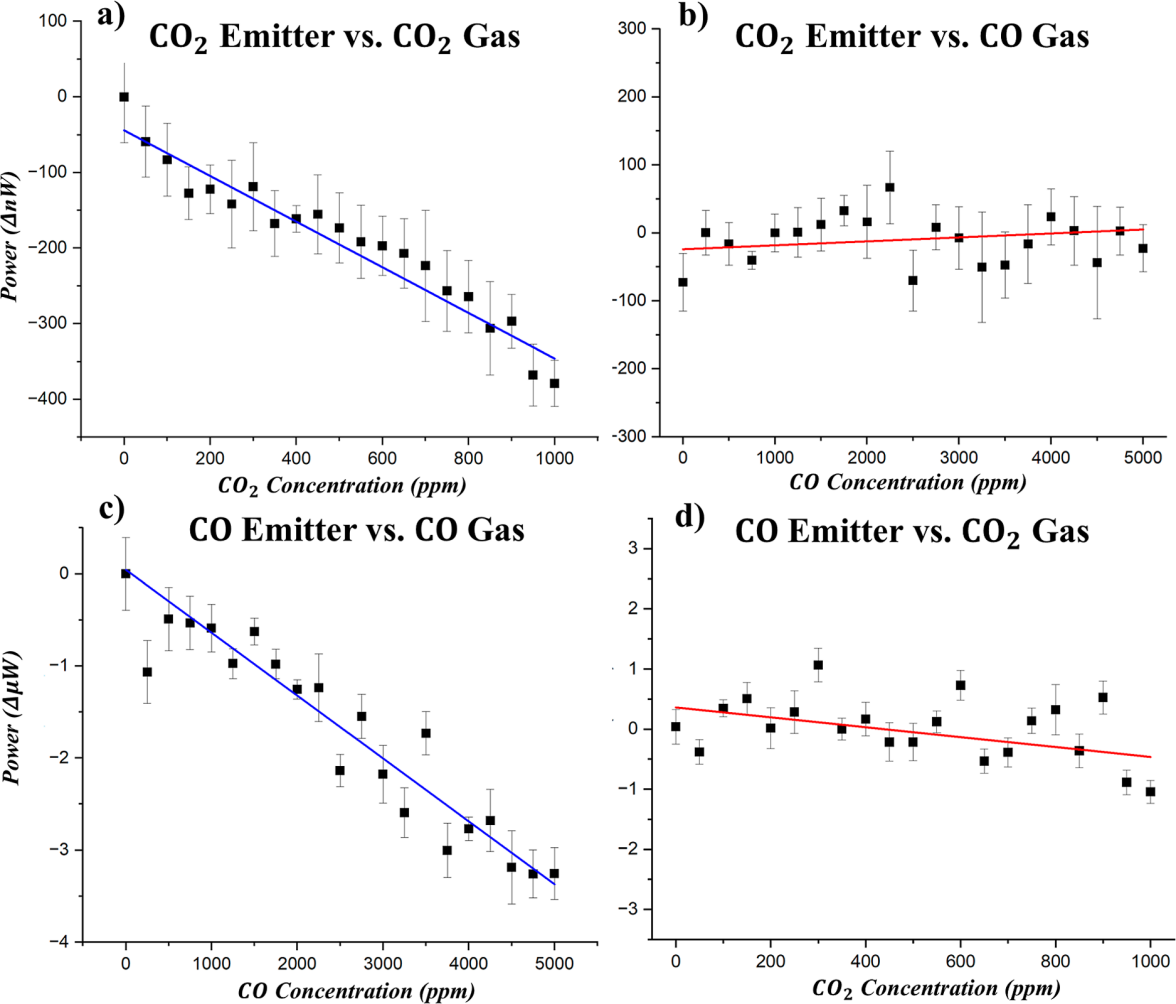
(a) Power absorption from the CO_2_ emitter at varying CO_2_ concentrations. (b) Power absorption from the CO_2_ emitter at varying CO concentrations. (c) Power absorption from the CO emitter at varying CO concentrations. (d) Power absorption from the CO emitter at varying CO_2_ concentrations.

Together, these results quantify the performance of inversely designed a-DBR emitters for filterless NDIR gas sensing. The dual-band C_3_H_8_ emitter enhanced detection sensitivity by up to 184% compared to a commercial single-band NDIR filter, while the CO and CO_2_ emitters exhibited Q-factors of 76.7 and 52.6, respectively, enabling clear spectral discrimination between closely spaced absorption features. Additionally, minimal angular dispersion and stable spectral alignment under thermal variation were observed across all emitters.

## Methods

### Emitter Fabrication

The multipeak thermal emitter was fabricated using radio frequency (RF) sputter deposition^51^ to deposit aperiodic multilayers of Ge and AlO_*x*_. Ge layers were deposited from a 2″ diameter Ge target in a pure Ar environment at a total pressure of 3 mTorr. AlO_*x*_ layers were reactively sputtered from a 2″ diameter Al target in a mixed 10% O_2_ and 90% Ar atmosphere at a total pressure of 5 mTorr. All Ge and AlO_*x*_ layers were deposited sequentially at ambient temperature without breaking the vacuum.

For the C_3_H_8_ emitter, the a-DBR was deposited on a low-resistivity Si substrate (ω_p_ ≈ 4.73 × 10^12^ rad·s^–1^). For the single-peak CO and CO_2_ emitters, an In-doped CdO layer with carrier concentration *N*_d_ = 3.85 × 10^20^ cm^–3^ was first grown by reactive high-power impulse magnetron sputtering (HiPIMS),^52^ following procedures described in previous reports.^34,36^ The three-layer a-DBR stacks were then deposited on the CdO film at ambient temperature using electron-beam evaporation from Ge (99.999%) and DyF_3_ (99.9%) sources. Layer thicknesses were monitored in real time using a quartz crystal microbalance throughout the deposition.

### Angle-Resolved Emissivity Measurements

Angle-resolved emissivity was measured using a Bruker Vertex 70v FTIR spectrometer^53^ coupled to a custom-built heated rotation stage. Spectra were collected from −60° to +60° in 5° increments, with the sample temperature held between 200 and 300 °C. Angular dispersion of each emission peak was quantified by fitting the peak frequency to a second-order Taylor^54^ expansion, as shown in eq 2:

2 $$\omega_{r,i}\left(\theta \right)=\omega_{0,i}+\frac{b_{i}}{2}\theta^{2}$$

where θ is the emission angle, ω_*r*,*i*_ is the resonant frequency, ω_0,*i*_ is the emission frequency along the surface normal, and *b*_*i*_ is the phenomenologically determined band curvature of mode *i*. The extracted *b*_*i*_ values were used to quantify the angular dispersion and evaluate spectral overlap with target absorption bands.

### Gas Sensing Measurements

Gas sensing measurements were performed using a custom-built NDIR setup (schematic in Supporting Information). The dual-peak a-DBR emitter was mounted on a Linkam heating stage^55^ and operated at 300 °C. Emission from the sample was collected using a Winston cone collimator positioned at the focal point to efficiently capture both normal and off-normal emissions. The collimated beam was directed through a 10 cm gas cell (model 162-10-CPF, Pike Technologies)^56^ equipped with CaF_2_ windows and then modulated with a mechanical chopper wheel. The modulated signal was detected with a pyroelectric detector, and the output was processed by using a lock-in amplifier to monitor changes in intensity. Gas concentrations were introduced into the cell by using controlled flow, and the resulting reduction in detected power was recorded and analyzed according to the Beer–Lambert behavior. Additional details, including concentration calibration and signal-averaging parameters are provided in the Supporting Information.

## Conclusion

This work demonstrates a filterless NDIR sensing strategy that leverages aperiodic distributed Bragg reflectors (a-DBRs) to achieve both high sensitivity and selectivity while minimizing fabrication complexity and cost. By applying gradient descent optimization, we designed and fabricated frequency-selective thermal emitters without the need for lithographic patterning or additional spectral filtering components.

We first validated this approach through the development of a multipeak emitter targeting both the C–H deformation and stretching modes of propane (C_3_H_8_). Simultaneous targeting of multiple absorption bands enabled enhanced sensitivity, particularly at low gas concentrations, and broadened the dynamic sensing range, illustrating the value of multiresonant designs in improving the detection performance. To further evaluate the spectral selectivity, we developed two single-peak, high-Q emitters for carbon monoxide (CO) and carbon dioxide (CO_2_), which possess vibrational features in close spectral proximity. Angle-resolved thermal emission measurements and gas sensing experiments confirmed strong spectral isolation and minimal crosstalk between the two targets, demonstrating the effectiveness of this approach for selective detection, even when analyte resonances are narrowly spaced.

Together, these results establish inversely designed a-DBRs as a promising path forward for compact, low-cost NDIR gas sensors. These planar emitters are inherently compatible with wafer-scale fabrication, enabling further cost reduction in commercial implementations, and when packaged using standard hermetic sealing approaches employed in commercial NDIR systems, the devices are expected to exhibit long-term stability comparable to existing thermal emitter technologies. By eliminating the need for filters, narrowband detectors, or lithographically patterned metasurfaces, this method breaks the inherent trade-off between sensitivity and selectivity found in conventional NDIR systems. This tunable and simple design could support a wide range of emerging gas sensing applications from environmental monitoring to medical diagnostics.

## Supplementary Material



## Notes

Temporal coupled-mode theory, stochastic gradient descent inverse design framework, angular dispersion modeling, dielectric functions of Ge, AlO_*x*_, and DyF_3_ at elevated temperatures, cross-sectional scanning electron microscopy analysis and layer thickness statistics of a-DBR stacks, band-dependent contributions to propane sensing, *k*-space emission maps, temperature-dependent emission stability, custom tabletop gas-sensing setup, Beer–Lambert law implementation, power and sensitivity calculations, and comparison to commercial NDIR gas sensors (PDF)
