# Supplementary material for: Multiresonant Nondispersive Infrared Gas Sensing: Breaking the Selectivity and Sensitivity Trade-Off
Source: ACS Photonics. 2026 Mar 12;13(7):1854–62. doi: 10.1021/acsphotonics.5c02787 (PMC13047729; doi:10.1021/acsphotonics.5c02787)
Supplement: Supplementary file 1 [file ph5c02787_si_001.pdf]

Supplementary Information

Multi-resonant non-dispersive infrared gas sensing: breaking the selectivity and sensitivity tradeoff

Emma R. Bartelsen[1,2], J. Ryan Nolen[3], Christopher R. Gubbin[3], Mingze He[2,4], Ryan W. Spangler[5], Joshua Nordlander[5], Cassandra L. Bogh[3], Katja Diaz-Granados[1], Simone De Liberato[3,6,7], Jon-Paul Maria[5], James R. McBride[8], Joshua D. Caldwell[1,2,3]

[1] Interdisciplinary Materials Science Program, Vanderbilt University, Nashville, Tennessee 37240, USA

[2] Department of Mechanical Engineering, Vanderbilt University, Nashville, Tennessee 37235, USA

[3] Sensorium Technological Labs, 6714 Duquaine Ct, Nashville, Tennessee 37205, USA

[4] Photonics Initiative, Advanced Science Research Center, City University of New York, New York, NY 10031, USA

[5] Department of Materials Science and Engineering, The Pennsylvania State University, University Park, Pennsylvania 16802, USA

[6] Istituto di Fotonica e Nanotecnologie, Consiglio Nazionale delle Ricerche (CNR), Piazza Leonardo da Vinci 32, Milano, 20133, Italy

[7] School of Physics and Astronomy, University of Southampton, University Road, Southampton, SO17 1BJ, United Kingdom

[8] Department of Chemistry, The Vanderbilt Institute of Nanoscale Science and Engineering, Vanderbilt University, Nashville, TN 37235, USA

S1. Temporal Coupled Mode Theory (TCMT)

Temporal coupled mode theory (TCMT) is a compact analytical framework used to describe how electromagnetic waves couple into and out of resonant modes in photonic structures.[1] In this approach, the resonant response is modeled by Lorentzian functions defined by the central frequency, linewidth, and the coupling strength to external channels. TCMT links the temporal dynamics of resonant modes with their absorptivity and emissivity spectra, making it well-suited for describing systems such as distributed Bragg reflector cavities, plasmonic resonances, and Tamm plasmon modes.

Here, TCMT was used to model the thermal emission spectra of the designed Tamm plasmon emitters and to estimate the power collected by the parabolic mirror in the experimental setup. The emission profiles were represented as Lorentzian resonances centered at frequencies extracted from experimental measurements, with linewidth and curvature parameters determined by fitting. For the CO and CO_2 emitters, a single Lorentzian resonance was sufficient, while the C_3H_8 emitter required two resonances to capture its multiple vibrational modes. This framework enabled calculation of angle- and frequency-dependent emissivity, incorporation of Planck's blackbody distribution at the emitter temperature, and angular integration to estimate the total collected power. These results provided a physically meaningful way to connect experimental measurements with modeled emission spectra.

S2. Stochastic gradient descent implementation for Tamm plasmon design

A stochastic gradient descent (SGD) inverse design framework, previously developed and published by our group[2], was used to optimize the distributed Bragg reflector (DBR) stacks supporting the Tamm plasmon resonances in this work. The framework integrates a differentiable transfer matrix method with the Adam optimizer (Adaptive Moment Estimation) to adjust layer thicknesses until the calculated resonance matches a user-defined spectral target.

The framework was applied to design multilayer Ge/DyF_3 stacks on doped CdO substrates for CO and CO_2 detection, and AlO_x/Ge stacks on Si substrates for C_3H_8 emission. Each design targeted narrowband transmission features centered at the vibrational frequencies of the corresponding gas. The algorithm outputs the optimized thickness profile, designed spectrum, and convergence history, which were subsequently compared with experimental measurements. The full implementation and example training scripts are available in Ref. 3[3], and a detailed description of the methodology is provided in Ref. 2[2].

S3. Variable angular dispersion modeling via Taylor expansion

The angular dispersion of the Tamm plasmon resonances was modeled using a second-order Taylor expansion around normal incidence ($\theta = 0$):

$$\omega_{r,i}(\theta) \approx \omega_{0,i} + \frac{1}{2} b_i \theta^2$$

Where $\omega_{r,i}$ is the resonance frequency at emission angle θ, $\omega_{0,i}$ is the resonance frequency at normal incidence, and b_i is the band curvature of mode i. This expansion provides an approximation of the resonance dispersion, reducing the angular dependence to a single curvature parameter. This form is well-suited to our emitters, as the TP modes exhibit smooth, symmetric dispersion about the surface normal. The fitted curvature values reported in the main text thus directly quantify the robustness of spectral overlap with C_3H_8, CO_2, and CO absorption across a broad angular range.

S4. Dielectric functions of Ge, AlO_x, and DyF_3 at 300°C

The dielectric functions of Ge, AlO_x, and DyF_3 at 300°C were used as input parameters for transfer matrix simulations of the a-DBR stacks. Experimental ellipsometry provided the tabulated datasets, giving the real (ε′) and imaginary (ε″) parts of the permittivity as a function of wavenumber (**Fig. S1**). From these values, the complex refractive index $\tilde{n} = n + ik = \sqrt{\varepsilon' + i\varepsilon''}$ was calculated and incorporated into the optimization and design framework.

For Ge, the dielectric response across the mid-infrared is weakly dispersive, with $\varepsilon' \approx 18 - 19$ and $\varepsilon'' \lesssim 10^{-2}$. This corresponds to a high refractive index (n ≈ 4.2) with negligible absorption (k ≈ 0), consistent with Ge being a low-loss, high-index material in this spectral window. In the CO and CO_2 operation windows, from 2100 to 2400 cm^{-1}, DyF_3 exhibits $\epsilon' \approx 2.838$ with $\epsilon'' \approx 0$, which results in n ≈ 1.68 and k ≈ 0. In the propane window near 2700 to 3000 cm^{-1}, AlO_x exhibits ϵ' approximately 2.3 to 2.5 with small ϵ'', which corresponds to n ≈ 1.5 to 1.6 and k ≈ 0. DyF_3 and AlO_x therefore serve as the low-index counterpart to Ge in their respective bands, with index contrasts relative to Ge of Δn ≈ 2 .5 for DyF_3 and Δn ≈ 2.6 to 2.7 for AlO_x, which is sufficient to open wide photonic bandgaps and to support narrowband Tamm plasmon modes.

The selection between DyF_3 and AlO_x reflects a balance between optical transparency and fabrication practicality. DyF_3 is more ideal optically because it is extremely transparent in the mid-infrared, but precise control of layer thickness is more difficult to achieve during sputtering. The CO and CO_2 emitters consisted of four layers arranged as CdO, Ge, DyF_3 and Ge. Using DyF_3 in for these a-DBR stacks was practical because only a single DyF_3 layer was

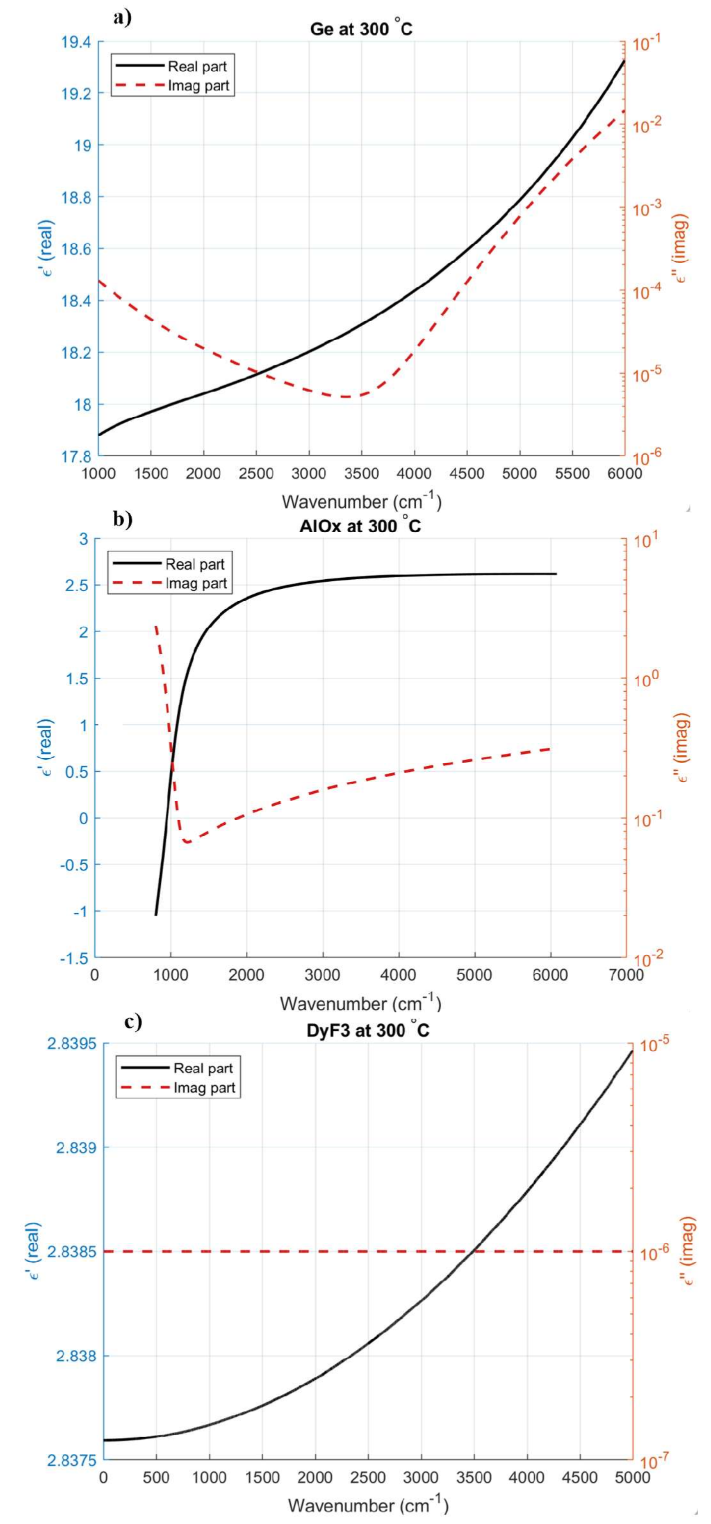

Figure S1. Real (ε′) and imaginary (ε″) parts of the dielectric function for (a) Ge, (b) AlOx, and (c) DyF_3 at 300 °C.

required. The dual emitter for propane required an eight-layer stack. In this case, using multiple layers of DyF_3 would have introduced greater cumulative error, since achieving nanometer-level thickness control with DyF_3 is more difficult than with AlO_x. To minimize the accumulation of deviations across the stack, AlO_x was selected as the low-index counterpart, prioritizing deposition reliability even though its losses are slightly higher.

S5. Target and as-grown layer thicknesses of a-DBR stacks

Accurate control of layer thickness is critical for achieving the designed spectral response of each stack, as small deviations can shift resonance positions or broaden the emission linewidths. To assess the fidelity and uniformity of the deposition process, cross-sectional SEM measurements were taken at multiple points across each structure. Values from four regions (**Figs. S2 and S4**) were measured and averaged to account for spatial variations across the wafer. From these measurements, the mean, median, standard deviation, and percent error were calculated relative to the target design (**Tables S1 and S2**), providing a quantitative evaluation of growth precision. Overall, the layer stacks closely match their target thicknesses, confirming uniform deposition, consistent material growth rates, and reproducible fabrication across samples.

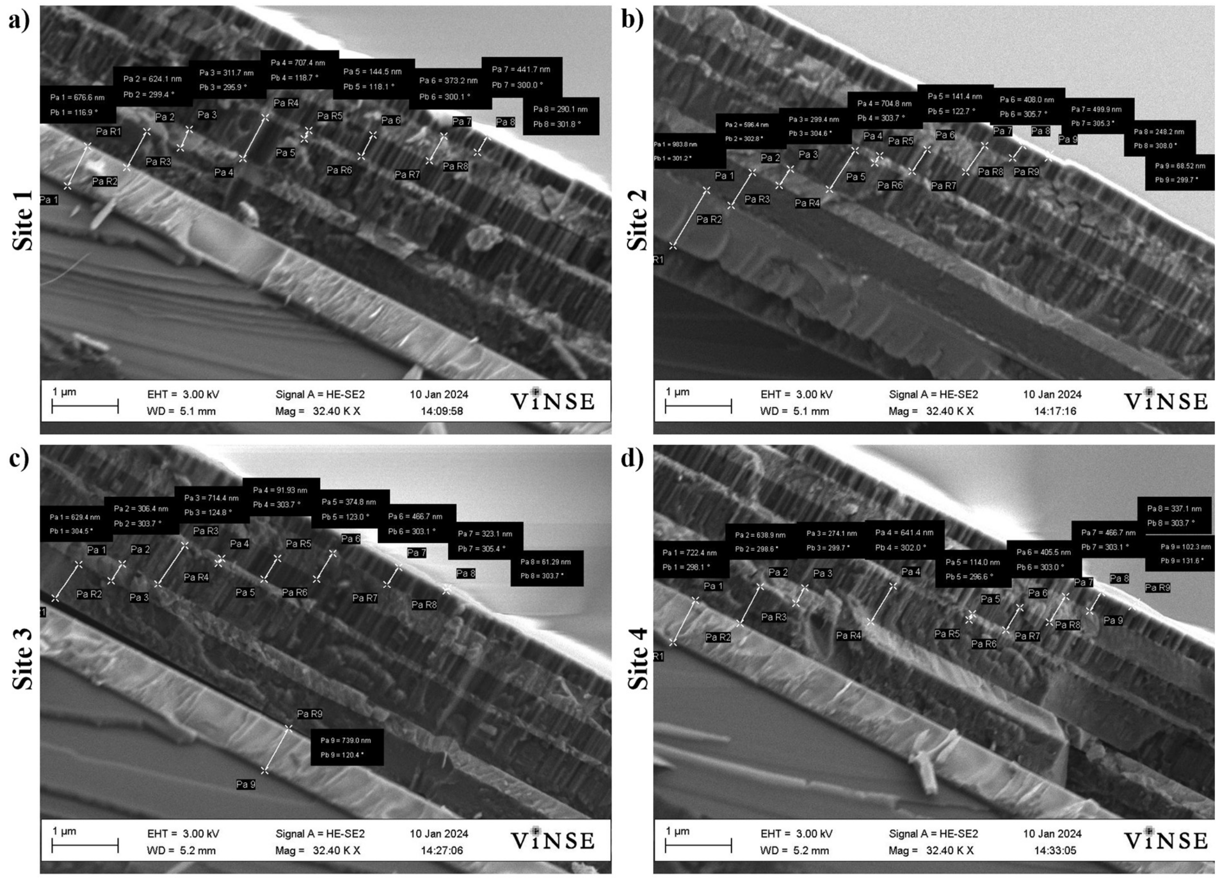

Figure S2. Cross-sectional SEM images of the C_3H_8 emitter at four regions: (a) Site 1, (b) Site 2, (c) Site 3, and (d) Site 4, showing consistent layer thicknesses and uniformity across the a-DBR stack.

C_3H_8 a-DBR

The C_3H_8 emitter consisted of eight alternating Ge and AlO_x layers deposited on Si. Cross-sectional SEM images from four regions of the wafer (**Figs. S2a – S2d**) were used to measure individual layer thicknesses. The expected and measured values are summarized in **Table S1**, with statistical results shown in **Fig. S3**.

Table S1. Expected and measured layer thicknesses for the C_3H_8 emitter obtained from SEM cross-sections at four regions (Site 1 – 4). Reported values include the mean, median, standard deviation (SD), absolute error, and percent error relative to the target design.

**C_3H_8 a-DBR Layer Thicknesses (nm)**										
Layer #	***Expected***	***Site 1***	***Site 2***	***Site 3***	***Site 4***	***Mean***	***Median***	***SD***	***Absolute Error (nm)***	***Percent Error (%)***
1 (Ge)	698	676.6	983.8	739.0	722.4	780.5	730.7	138.1	82.45	11.81
2 (AlO_x)	612	624.1	596.4	629.4	638.9	622.2	626.8	18.26	10.20	1.667
3 (Ge)	303	311.7	299.4	306.4	274.1	297.9	302.9	16.65	5.100	-1.683
4 (AlO_x)	670	707.4	704.8	714.4	641.4	692.0	706.1	33.98	22.00	3.284
5 (Ge)	108	144.5	141.4	91.93	114.0	123.0	127.7	24.81	14.96	13.85
6 (AlO_x)	382	373.2	408.0	374.8	405.5	390.4	390.2	18.95	7.375	1.926
7 (Ge)	453	441.7	499.9	466.7	466.7	468.8	466.7	23.88	15.75	3.477
8 (AlO_x)	297	290.1	248.2	323.1	337.1	299.6	306.6	39.54	2.625	0.884
Average								39.27	20.06	4.402

Across all layers, the mean deviation from the design was 4.4%, with the largest variation (13.9%) occurring in the thinnest Ge layer (≈ 100 nm), which is more sensitive to rate fluctuations during deposition. The average absolute error was 39.27 nm, demonstrating close agreement between designed and fabricated values. These results confirm that the layer thickness precision achieved is sufficient to maintain the intended resonance behavior of the structure.

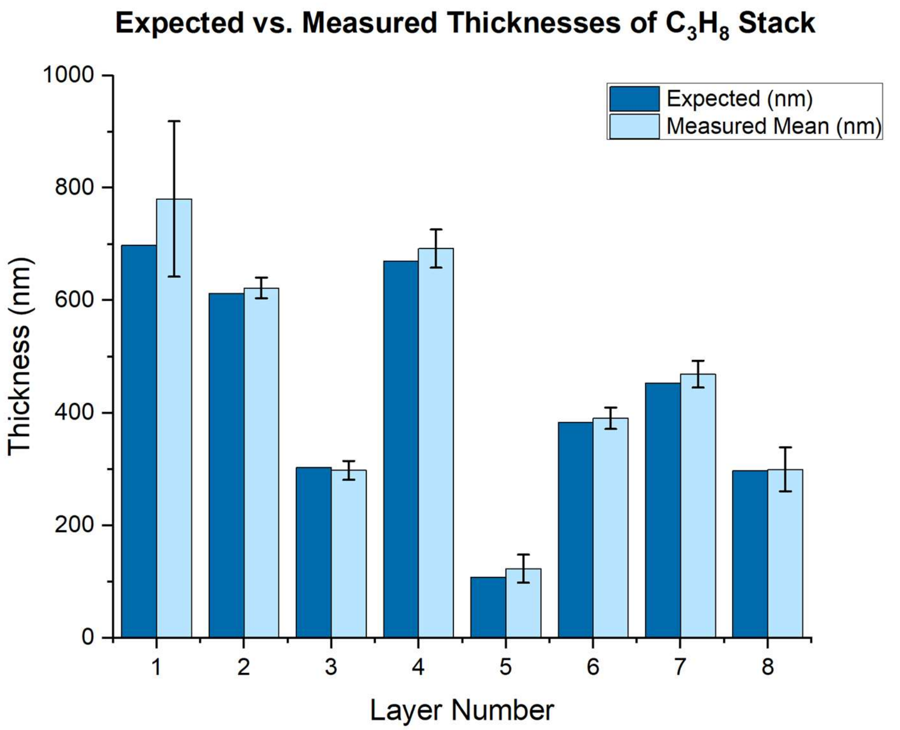

Figure S3. Comparison of expected and measured layer thicknesses for the C_3H_8 emitter. Bars represent target (dark blue) and measured mean (light blue) values, with error bars showing SD across four regions. All expected values fall within the SD of the measurements, confirming strong agreement with design targets.

CO a-DBR

The CO and CO_2 emitters were grown on a doped CdO substrate, creating challenges in SEM imaging due to surface charging under the electron beam. To mitigate this, a 100 nm Au

overlayer was deposited by resistive thermal evaporation to provide a conductive path and reduce charging. Prior to cross-sectioning, each region of interest was capped with a Pt layer deposited in situ using electron-beam-assisted deposition to protect the surface during focused ion beam (FIB) milling. The cross-sections were then prepared using a Ga^+ ion beam to expose the layer structure for high-resolution SEM imaging (**Fig. S4**).

SEM cross-sections from four regions (Figs. S4a–S4d) were analyzed to extract as-grown layer thicknesses. The results are summarized in **Table S2** and plotted in **Fig. S5**. The CO emitter exhibited an average percent error of 11.5%, with the smallest deviation (1.6%) in the ≈ 480 nm AlO_x layer and the largest (24.4%) in the ≈ 600 nm Ge layer. The average absolute error was ≈ 50 nm. Despite these deviations, the relative thickness ratios remained within the tolerances required to maintain the intended optical interference and spectral response. The CO_2 emitter was processed and analyzed using the same methodology, and the results reflected similar trends to those observed for the CO emitter.

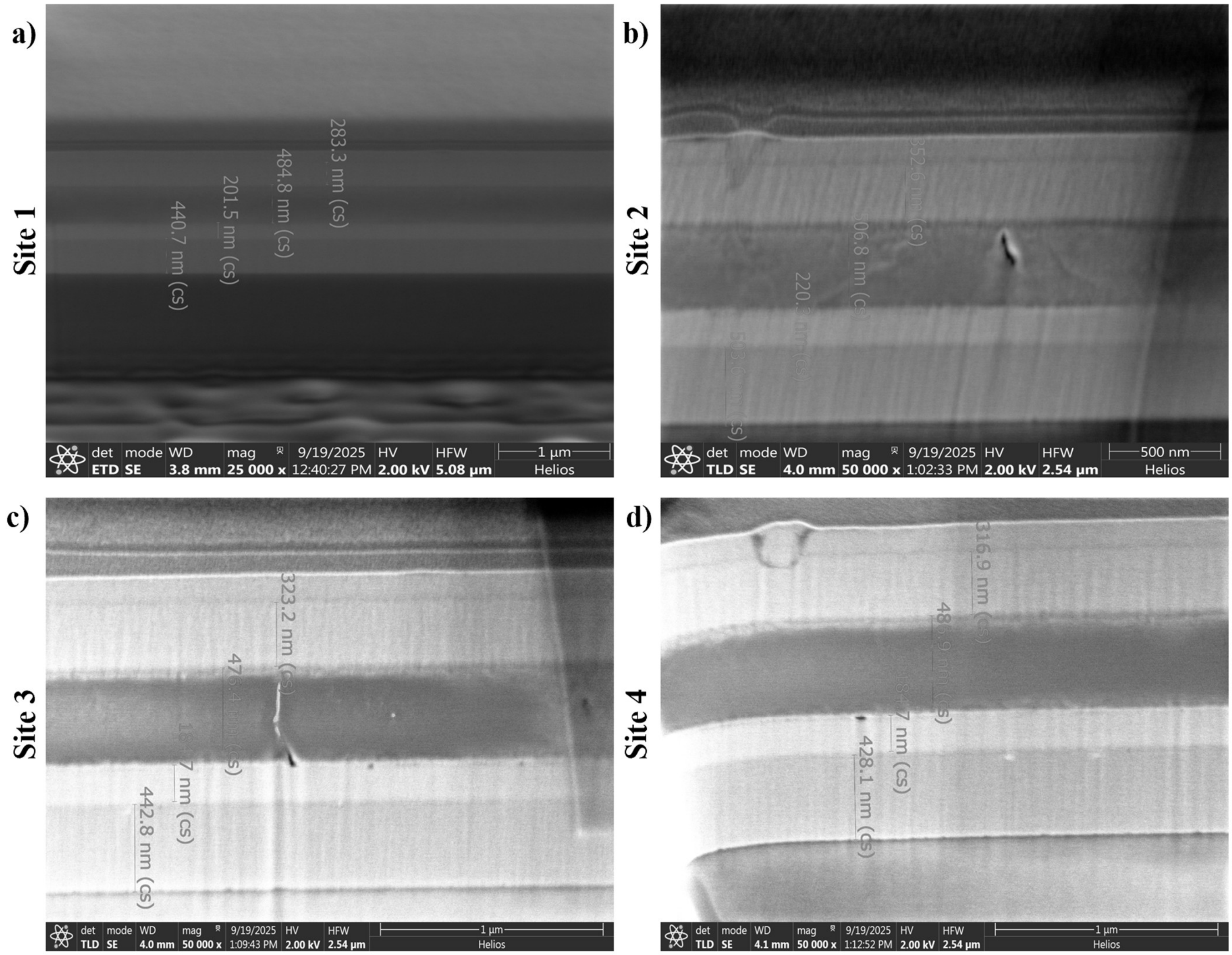

Figure S4. Cross-sectional SEM images of the CO emitter obtained from FIB-prepared cross-sections at four regions: (a) Site 1, (b) Site 2, (c) Site 3, and (d) Site 4. Images show consistent layer thicknesses and uniformity across the a-DBR stack.

Although the CdO layer deviated from its target thickness, this variation does not significantly affect device performance. Once the CdO film exceeds approximately 300 nm, it behaves as an optically thick, highly reflective substrate in the mid-infrared. At this thickness, the electromagnetic field penetration depth is much smaller than the film thickness, and the substrate effectively acts as a semi-infinite reflector. Thus, moderate deviations in CdO thickness do not alter the resonance condition or overall emission characteristics. Overall, the observed layer thickness deviations across all emitter types are on the order of the expected ±10% range typically associated with laboratory-scale sputtered multilayer structures, indicating reproducible fabrication at the research scale.

Table S2. Expected and measured layer thicknesses for the CO emitter obtained from FIB-SEM cross-sections at four regions (Site 1–4). Reported values include the mean, median, standard deviation (SD), absolute error, and percent error relative to the target design.

**CO a-DBR Layer Thicknesses (nm)**										
Layer #	***Expected***	***Site 1***	***Site 2***	***Site 3***	***Site 4***	***Mean***	***Median***	***SD***	***Absolute Error (nm)***	***Percent Error (%)***
1 (Ge)	295	283.3	352.6	323.2	316.9	319.0	320.1	28.43	24.00	8.14
2 (DyF_3)	481	484.8	506.8	476.4	486.9	488.7	485.9	18.26	7.73	1.61
3 (Ge)	177	201.5	220.3	184.7	184.7	197.8	193.1	16.65	20.80	11.75
4 (CdO)	600	440.7	503.6	442.8	428.1	453.8	441.8	33.98	146.20	24.37
Average								23.03	49.68	11.46

S6. Band-Dependent Contributions to Propane Detection

Simulated transmission spectra were generated to evaluate how the two emission bands of the dual-band C_3H_8 emitter contribute to propane sensing across varying gas concentrations. **Figure S6** presents a false-color map of the simulated spectral response as a function of wavenumber and propane concentration, where yellow indicates stronger absorption and blue corresponds to minimal absorption or higher transmission.

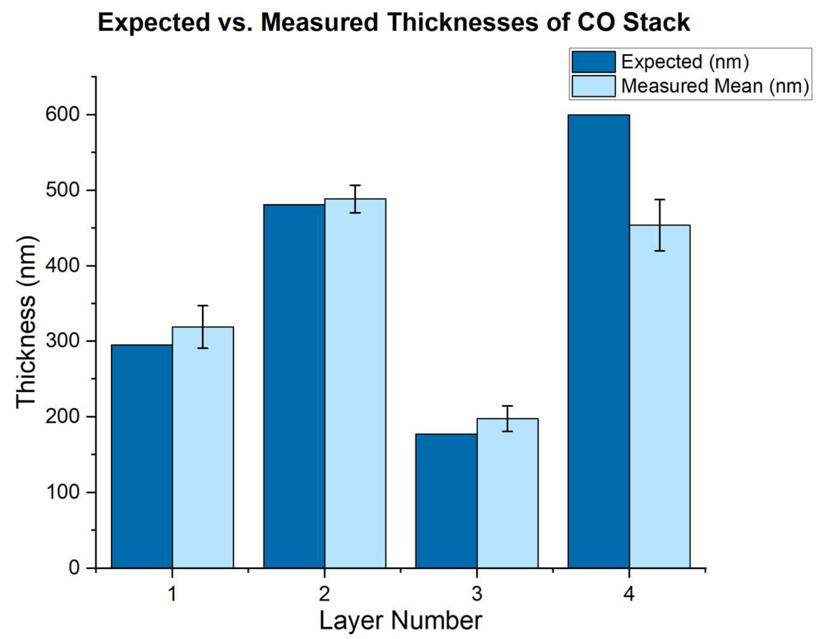

Figure S5. Comparison of expected and measured layer thicknesses for the CO emitter. Bars represent target (dark blue) and measured mean (light blue) values, with error bars showing SD across four regions. All expected values fall within the SD of the measurements except for the CdO layer (Layer 4).

Two primary absorption features are observed near 2768 cm^{-1} and 1420 cm^{-1}, corresponding to the C–H stretching and C–H bending vibrational modes of propane, respectively. As the propane concentration increases from 0% to 100%, both bands exhibit progressively stronger absorption, consistent with increased interaction between the

emitted light and the target gas. However, each resonance displays a distinct sensitivity profile with concentration.

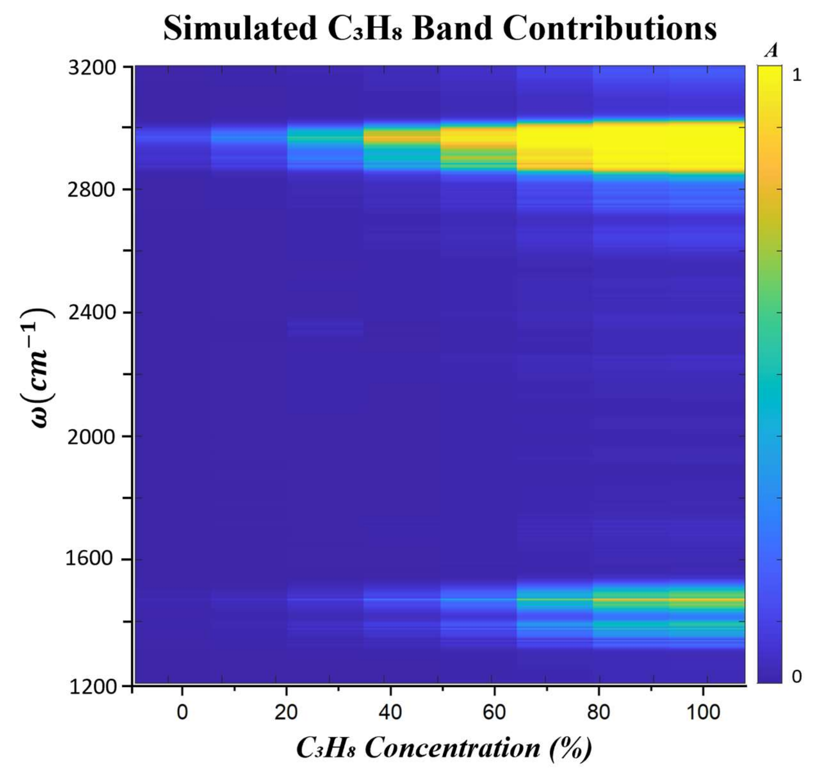

Figure S6. Simulated absorptance map illustrating concentration-dependent absorption at the 1420 cm⁻¹ and 2768 cm⁻¹ bands.

At low propane concentrations (below approximately 30%), the upper band at 2768 cm⁻¹ dominates the response due to its higher absorption coefficient, producing a rapid increase in absorption intensity. As concentration increases beyond this point, this band begins to saturate, where additional propane produces minimal further absorption. The lower band at 1420 cm⁻¹, however, continues to vary with concentration, maintaining a measurable response even after the upper band has saturated.

This complementary behavior between the two emission bands extends the overall dynamic range of the sensor. The upper band provides high sensitivity at low concentrations, while the lower band maintains responsiveness at elevated concentrations where the upper band becomes saturated. Together, these bands produce a continuous and quantifiable sensing response across the full concentration range, a capability not achievable using a single-band emitter.

S7. k-Space Mapping of Dual-Band Emission

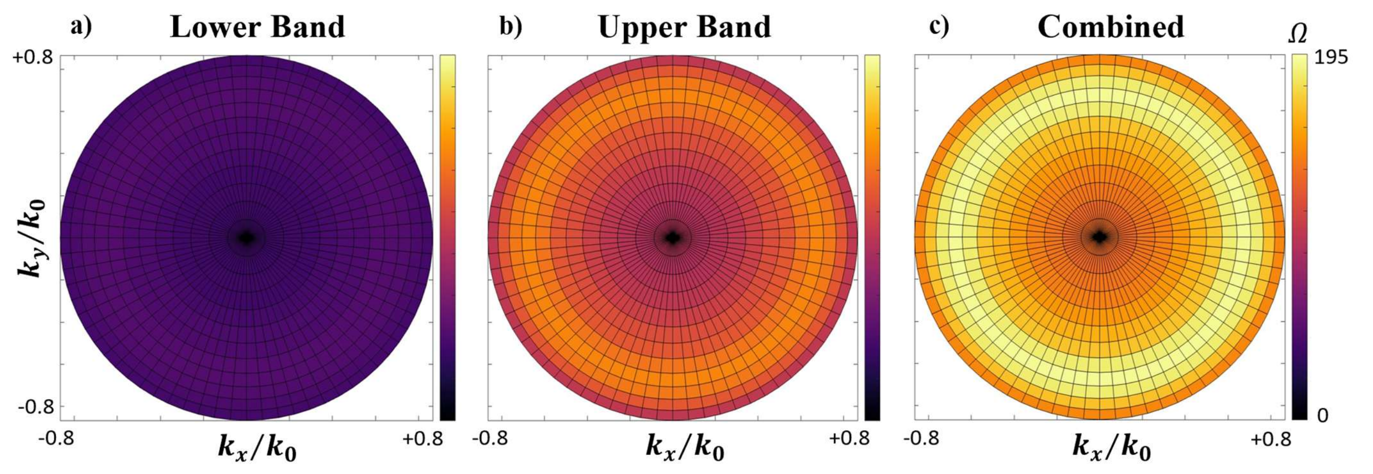

Figure S7. Simulated k-space maps showing the angular emission intensity of the dual-band C_3H_8 emitter for (a) the lower band at 1420 cm⁻¹, (b) the upper band at 2768 cm⁻¹, and (c) the combined emission.

Momentum-resolved emission simulations were performed to visualize the spatial confinement and angular distribution of the two resonant modes supported by the dual-band C_3H_8 a-DBR emitter. **Figures S7(a - c)** show the normalized emission intensity as a function of the in-plane wavevectors (k_x/k_0 and k_y/k_0) for the lower band, upper band, and their combined response, respectively. The color scale (Ω) represents the normalized emission strength across momentum space.

Both the lower (1420 cm⁻¹) and upper (2768 cm⁻¹) bands exhibit strong confinement within the light line, confirming that the observed features correspond to surface-confined optical modes rather than freely propagating radiation. This behavior reflects the hybrid nature of the optical states, which arise from coupling between the photonic cavity and metallic interface.

The circular symmetry of the intensity distributions indicates that the emission is spatially isotropic in-plane, consistent with the planar geometry of the a-DBR structure. While previous figures characterized the spectral dependence of the emission, these momentum-space maps provide complementary spatial information, showing how the radiative intensity varies with in-plane momentum. The combined map (**Fig. S7c**) demonstrates that the dual-band a-DBR design enhances the overall optical density of states and broadens the accessible angular range of emission, both of which facilitate stronger coupling and improved detection efficiency.

S8. Temperature-Dependent Emission Stability

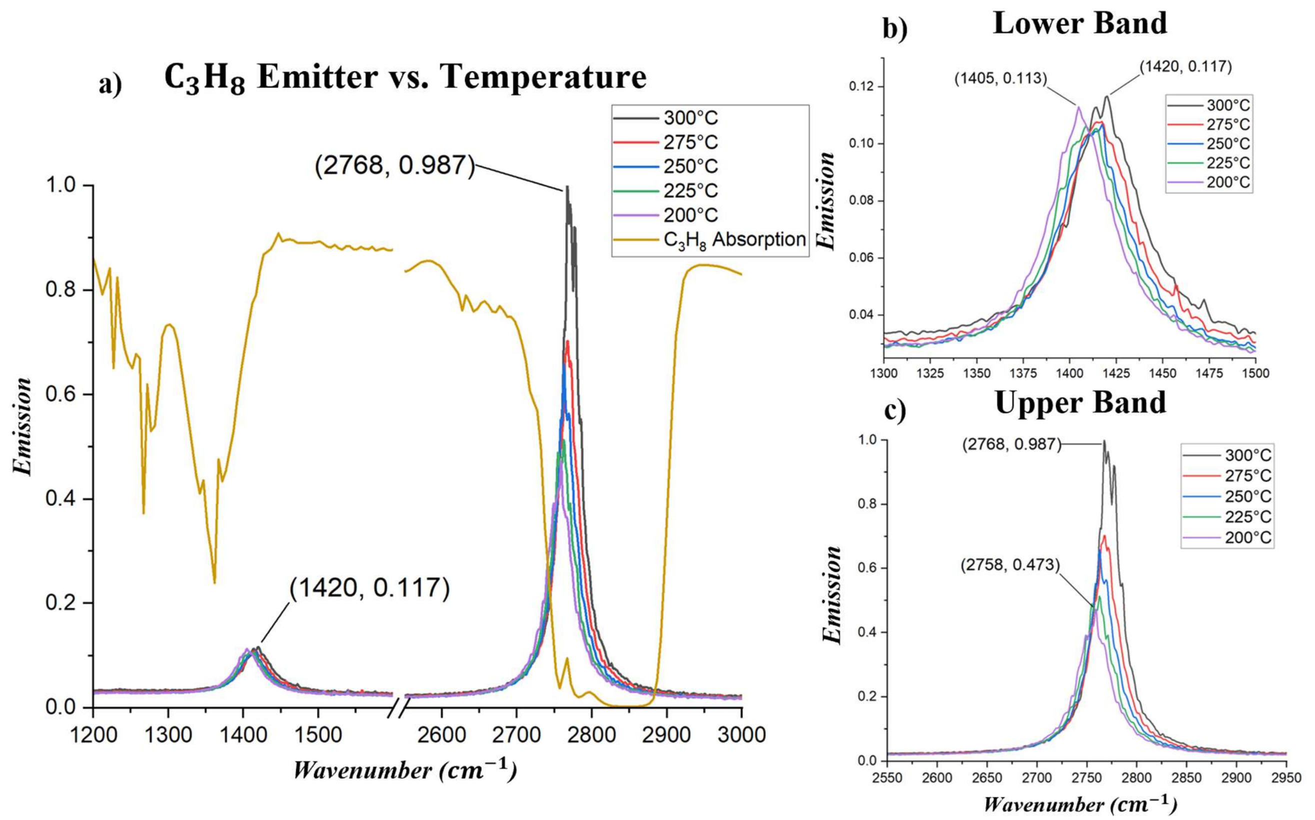

Figure S8. (a) FTIR emission measurements of the dual-band C_3H_8 emitter from 200–300°C. The lower band (b) centered near 1420 cm⁻¹ exhibits a 1.1% spectral shift, while the upper band (c) near 2768 cm⁻¹ shifts by 0.36%, confirming stable emission across the operating range.

Temperature-dependent emission spectra were collected for the CO, CO_2, and C_3H_8 emitters using Fourier-transform infrared (FTIR) spectroscopy and a Linkam heating stage. Emission measurements were performed from 200°C to 300°C in 25°C increments, allowing evaluation of thermal stability and resonance shifting with temperature.

Figures S8(b) and **S8(c)** show expanded views of the lower (1420 cm^{-1}) and upper (2768 cm^{-1}) emission bands of the dual-band C_3H_8 emitter presented in **Figure S8(a).** Across all devices, the emission peaks exhibit only minor spectral shifts with increasing temperature, confirming excellent thermal stability of the emitters within the operational range. The magnitude of each shift was quantified using the relative change in peak frequency between 200°C and 300°C, normalized to the average resonance frequency:

$$\text{Percent shift} = \frac{|\tilde{\nu}_{300} - \tilde{\nu}_{200}|}{\tilde{\nu}_{\text{avg}}} \times 100$$

where $\tilde{\nu}_{300}$and $\tilde{\nu}_{200}$are the resonance frequencies at 300°C and 200°C, respectively.

For the C_3H_8 emitter, the lower-energy band shifted from 1405 cm^{-1} at 200°C to 1420 cm^{-1} at 300°C, corresponding to a 1.1% shift, while the upper-energy band shifted from 2758 cm^{-1} to 2768 cm^{-1}, a 0.36% shift. Both resonances remain well aligned with the absorption bands of propane throughout the entire temperature range.

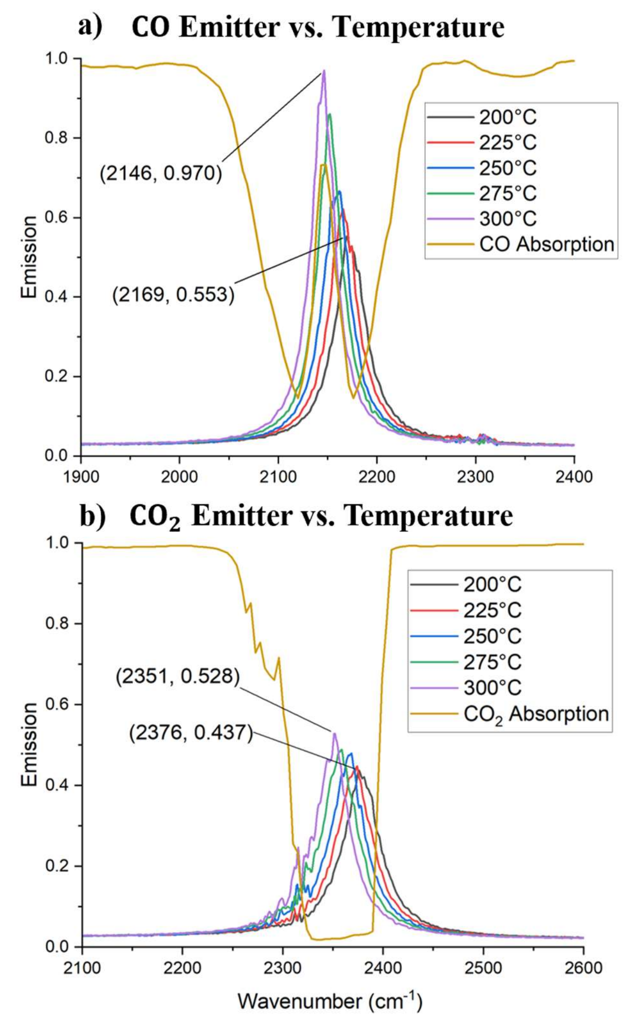

Figure S9. (a) Emission spectra of the CO emitter measured from 200 °C to 300 °C. (b) Emission spectra of the CO_2 emitter measured from 200 °C to 300 °C.

The CO emitter shifted from 2169 cm^{-1} at 200°C to 2146 cm^{-1} at 300°C, corresponding to a 1.1% shift, while the CO_2 emitter shifted from 2376 cm^{-1} to 2351 cm^{-1}, also a 1.1% shift as seen in **Fig. S9**. These small shifts indicate that the emission frequencies remain spectrally confined within the respective gas absorption bands, ensuring consistent spectral overlap during sensing operation.

Overall, all emitters display less than 1.1% spectral variation across the 200°C – 300°C temperature range. This minimal shift confirms that the emitters are thermally stable and maintain spectral alignment with the target gas absorption bands under typical operating conditions.

S8. Custom Tabletop Setup for Gas Sensing Measurements

A custom optical setup was constructed to measure gas absorption using emission from the aperiodic distributed Bragg reflector (a-DBR) thermal emitters as seen in **Fig. S10.** The emitters were mounted on a Linkam THMS600 heating stage (Linkam Scientific Instruments, UK)[4] to enable controlled temperature

operation. A custom gold-coated Winston cone (Optiforms, Inc.)[5] was positioned directly above the emitter to collimate the emitted radiation. The Winston cone featured an inner aperture of 8.0 mm, an entrance aperture (outer diameter) of 26.3 mm, and a parabolic reflective surface with a focal length of 7.9 mm. The a-DBR emitter was placed at the cone's focal point to ensure efficient collection and collimation of the emitted light.

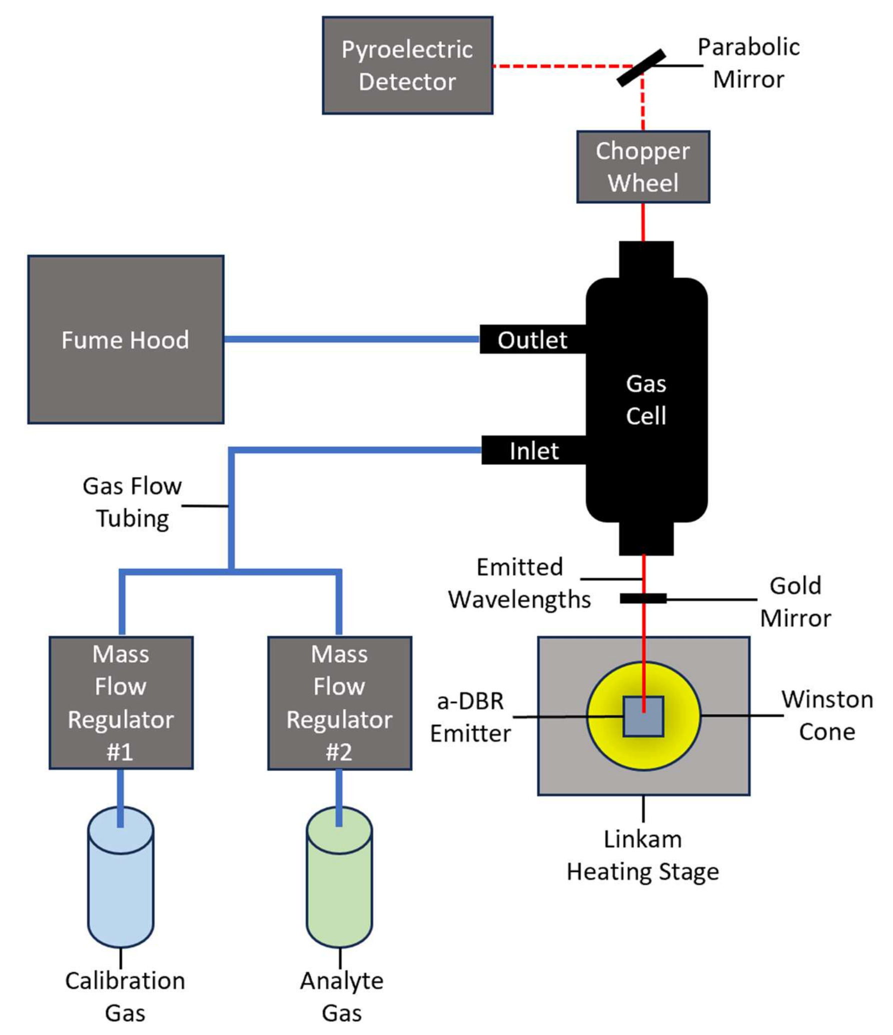

Figure S10. Schematic of the custom tabletop gas-sensing setup.

A gold-coated mirror set at 45° incidence deflected the axial (z-direction) emission by 90°, redirecting it horizontally along the gas-cell optical axis. The collimated emission then entered a 10 cm gas cell equipped with CaF_2 windows (transmission range ≈ 50,000 cm^{-1} – 800 cm^{-1})[6]. The gas cell was connected to two Alicat mass-flow controllers that regulated the flow of calibration and analyte gases.[7] A 99.999% pure N_2 calibration gas and the target analyte gas were mixed in controlled ratios to achieve the desired concentration levels.

The gas cell was initially purged with N_2 for 3 minutes prior to measurement. The flow ratio was then adjusted in 5% increments, beginning at 0 sccm analyte/200 sccm N_2 and increasing to 200 sccm analyte/0 sccm N_2. Each new concentration was allowed to flow for 30 seconds before recording a 60-second emission measurement. All gases were delivered through Tygon gas-transfer tubing secured with Prestolok fittings, and exhaust gases were safely vented into a fume hood.

After transmission through the gas cell, the emission passed through a mechanical chopper wheel, which modulated the continuous signal prior to detection. The modulated beam was then focused by a 1-inch diameter, 90° off-axis parabolic mirror with a protected silver coating and a reflected focal length (RFL) of 6 inches[8], directing the light onto a pyroelectric detector[9] positioned at the mirror's focal distance. The pyroelectric detector output (in watts) corresponded to the transmitted emission intensity and was recorded at a sampling rate of approximately 15 measurements per second, enabling quantification of gas concentration via Beer's law.

S9. Beer Lambert Law

The Beer–Lambert law describes the exponential attenuation of light as it passes through an absorbing medium, relating the transmitted intensity to the concentration and optical path length of the absorber. It is expressed as:

$$I = I_0 e^{-\alpha c L}$$

where I_0 and I are the incident and transmitted intensities, respectively, α is the absorption coefficient of the analyte, c is the concentration, and L is the optical path length. In logarithmic form, the absorbance A is given by:

$$A = \log_{10}(\frac{I_0}{I}) = \varepsilon c L$$

where ε is the molar absorptivity, a material- and wavelength-dependent constant.

In this work, the Beer–Lambert law was applied to quantify gas concentration from the measured emission intensity transmitted through the 10 cm gas cell. The pyroelectric detector measured the transmitted power (I) at each analyte concentration, while the initial power in pure N_2 (I_0) served as the reference intensity. The resulting absorbance spectra were then computed from the ratio I_0/I, allowing the relative change in transmitted intensity to be directly correlated to the analyte concentration. This relationship enabled quantitative assessment of gas absorption for CO, CO_2, and C_3H_8 using the a-DBR emitter as the tunable radiation source.

S10. Power calculations

A semi-analytical framework was developed to estimate the concentration-dependent transmitted power and minimum detection limit of the gas-sensing setup. The calculation incorporates the molar absorptivity of the target gas, the temperature- and angle-dependent emissivity of the emitter, the collection efficiency of the optical system, and the responsivity and noise characteristics of the detector.

Molar Absorptivity

The molar absorptivity (ε) of each gas species was derived from transmission spectra using the Beer–Lambert law:

$$-T = \varepsilon L c$$

where T is the measured transmission, L is the gas-cell path length (10 cm), and c is the gas concentration. From these measurements, ε was determined and used to estimate absorbance as a function of concentration and optical path length.

Emitter Radiated Power

The spectral power radiated by the a-DBR emitter was modeled as:

$$P_r = \int_0^{2\pi} \int_0^{\phi_{\max}} \int_{\lambda_0}^{\lambda_{\max}} I_{\mathrm{BB}}(T, \lambda)\, \varepsilon(\lambda, \theta) \sin\theta \cos\theta \, d\lambda \, d\theta \, d\phi$$

where $I_{\mathrm{BB}}(T, \lambda)$ is the Planck blackbody intensity, and $\varepsilon(\lambda, \theta)$ is the angle-dependent emissivity obtained experimentally via FTIR emission measurements. The limits $\phi_{\max}$ and $\lambda_{\max}$ are determined by the geometry and optical bandwidth of the Winston cone and gas cell, respectively.

For an ideal blackbody,

$$I_{\mathrm{BB}}(T, \lambda) = \frac{2hc^2}{\lambda^5}\left(e^{\frac{hc}{\lambda k_B T}} - 1\right)^{-1}$$

and the total radiated power is modulated by the emitter's emissivity spectrum $\varepsilon(\lambda, \theta)$.

Optical Transfer Function

Optical throughput was modeled via a multiplicative transfer function representing reflections and transmissions through all optical components:

$$\tau(\lambda, c) = C\, R_{m1}\, T_{w1}(\lambda)\, T_{gc}(\lambda, c)\, T_{w2}(\lambda)\, R_{bs}\, R_{p1}$$

where R_m and T_w denote reflection and transmission coefficients of the mirrors and windows, respectively, and $T_{gc}(\lambda, c)$ represents gas-cell transmission at concentration c. The calibration constant C accounts for unmodeled DC offsets and normalization to experimental data. The total detected power is then expressed as:

$$P_d(c) = \int_0^{2\pi} \int_0^{\phi_{\max}} \int_{\lambda_0}^{\lambda_{\max}} I_{\mathrm{BB}}(T, \lambda)\, \varepsilon(\lambda, \theta)\, \tau(\lambda, c) \sin\, \theta \cos\, \theta\, d\lambda\, d\theta\, d\phi$$

Detector Noise and Sensitivity

The pyroelectric detector used in this work (Ophir Optics RM9, with lock-in amplifier)[9] has a noise-equivalent power (NEP) of approximately 95 nW·Hz$^{-1/2}$, derived from the manufacturer's specified power noise level of 30 nW averaged over a 10 s interval. The effective detection range of the system is 100 nW to 100 mW, with an 8 mm aperture diameter. The minimum detectable signal ($P_{\min}$) was estimated by equating the concentration-dependent differential power change (ΔP_d) to the detector NEP, providing an estimate of the minimum detectable gas concentration under the experimental conditions.

S11. C_3H_8 Emitter Comparison to Commercial NDIR Gas Sensor

This section evaluates the enhancement in sensitivity achieved by the dual-band C_3H_8 aperiodic distributed Bragg reflector (a-DBR) emitter compared to a commercial single-band narrowband infrared (NDIR) gas sensor. The a-DBR emitter was specifically designed to provide simultaneous emission at two spectrally tailored resonances that align with the mid-infrared absorption features of propane (C_3H_8) near 2768 cm^{-1} and 1420 cm^{-1}, enabling direct comparison of dual- versus single-band performance.

The commercial InfraTec NDIR filter is centered at 2679 cm^{-1} (3.73 μm) with a full width at half maximum (FWHM) of 63.5 cm^{-1} and a peak transmission of 0.80.[10] In contrast, the custom a-DBR emitter exhibits dual-band emission centered at 2768 cm^{-1} and 1420 cm^{-1}, with FWHM values of 29.5 cm^{-1} and 61.2 cm^{-1}, respectively, and corresponding peak emissivities of 0.987 and 0.117.

To quantify the relative sensitivity, the detected power was modeled as the source spectrum weighted by the gas transmission,

$$T(\tilde{\nu}; C, L) = \exp\left[-\kappa(\tilde{\nu})\, C\, L\right] \approx 1 - \kappa(\tilde{\nu})\, C\, L,$$

where $\kappa(\tilde{\nu})$ is the propane absorption coefficient, C is concentration, and L is the gas-cell path length (10 cm). The fractional power change was then expressed as:

$$\frac{\Delta P}{P_0} \approx \int W(\tilde{\nu})\, \kappa(\tilde{\nu})\, d\tilde{\nu}\, C\, L = S\, C\, L,$$

where $W(\tilde{\nu})$ represents either the emitter emissivity or the InfraTec filter transmission spectrum. The proportionality constant S defines the spectral sensitivity, and the enhancement ratio was calculated as:

$$E = \frac{S_{\text{a-DBR}}}{S_{\text{InfraTec}}}.$$

Two cases were evaluated to examine the impact of the emitter's enhanced emissivity and its dual-band emission capability. The single-band enhancement compares emission near 2768 cm^{-1} to the InfraTec filter response, isolating improvements arising from the stronger and more spectrally matched emissivity of the a-DBR design. The dual-band enhancement, on the other hand, demonstrates the core advancement of this work, the ability to emit at two distinct resonance frequencies simultaneously, enabling multi-band detection within a single emitter and expanding sensing capability beyond what is possible with traditional single-band NDIR filters.

Using the InfraTec filter parameters (center = 2679 cm^{-1}, FWHM = 63.5 cm^{-1}), the integrated sensitivities were:

$$S_{\text{InfraTec}} = 8.7588, \qquad S_{\text{a-DBR 2768 band}} = 8.0967, \qquad S_{\text{a-DBR dual-band}} = 11.9565$$

yielding

$$E_{\text{single-band}} = 0.92, \qquad E_{\text{dual-band}} = 1.36$$

In this configuration, the commercial filter encompasses a broader spectral region, capturing a larger portion of the propane absorption band and therefore exhibiting inherently higher apparent sensitivity. However, this increased bandwidth also reduces chemical selectivity by broadening the detection window, increasing the likelihood of false positives from nearby gas species that are spectrally close.

To ensure a fair and physically consistent comparison, the InfraTec filter was recalculated using a matched FWHM of 29.5 cm^{-1}, equivalent to the linewidth of the a-DBR emitter's 2768 cm^{-1} resonance. This bandwidth matching normalizes the comparison so that the calculated enhancement reflects differences in emitter design rather than filter width. Under these matched conditions,

$$S_{\text{InfraTec, matched FWHM}} = 4.2090, S_{\text{a-DBR 2768 band}} = 8.0967, \qquad S_{\text{a-DBR dual-band}} = 11.9565$$

producing enhancement ratios of

$$E_{\text{single-band, matched FWHM}} = 1.92, \qquad E_{\text{dual-band, matched FWHM}} = 2.84$$

These results confirm that the single-band C_3H_8 a-DBR emitter already provides nearly a 1.92× improvement in sensitivity due to its higher emissivity, while the dual-band configuration further increases this enhancement to 2.84× by enabling simultaneous emission at multiple resonances within a single device. Together, these findings demonstrate that the a-DBR architecture enables a new class of multi-band emitters that improve sensitivity without compromising spectral selectivity, achieving performance beyond that of conventional NDIR systems.

S12. CO Emitter Comparison to Commercial NDIR Gas Sensor

This section evaluates the sensitivity of the spectrally selective CO aperiodic distributed Bragg reflector (a-DBR) emitter relative to another InfraTec narrowband infrared (NDIR) filter for CO gas detection.[11] Unlike the C_3H_8 emitter, the CO and CO_2 emitters were not designed to maximize sensitivity enhancement, but rather to demonstrate precise spectral selectivity and enable independent detection of spectrally similar gas absorption features. The calculated sensitivity enhancement is reported for completeness, but is not the primary design objective of these emitters.

The custom CO a-DBR emitter exhibits a single emission resonance centered at 2146 cm^{-1}, corresponding to the fundamental vibrational absorption band of CO. The emission peak has a FWHM of 27.54 cm^{-1} and a peak emissivity of 0.970. For comparison, the commercial InfraTec CO NDIR filter is centered near 2146 cm^{-1} with a FWHM of 83 cm^{-1} and a peak transmission of 0.86.

To allow for a more direct comparison of the two systems, two sensitivity analyses were performed. In the first case, the InfraTec filter response was recalculated using a matched FWHM of 27.54 cm^{-1}, equivalent to the linewidth of the a-DBR emitter. As in Section S.11, the detected power was modeled as the source spectrum weighted by the gas transmission, and the spectral sensitivity S was extracted from the linearized Beer–Lambert relation. Under matched-bandwidth conditions, the integrated sensitivities were found to be:

$$S_{\text{CO InfraTec, matched FWHM}} = 0.01773, \qquad S_{\text{CO a-DBR, matched FWHM}} = 0.01989.$$

The resulting enhancement ratio is:

$$E_{\text{CO, matched FWHM}} = 1.1218,$$

corresponding to a 12.2% increase in sensitivity relative to the commercial InfraTec CO NDIR filter. This enhancement arises primarily from the higher peak emissivity of the a-DBR emitter compared to that of the InfraTec filter within a spectrally constrained bandwidth.

In the second case, sensitivities were calculated using the unmatched bandwidths of each component to reflect their performance under typical operating conditions. Using the InfraTec filter FWHM of 83 cm^{-1} and the a-DBR emitter FWHM of 27.54 cm^{-1}, the extracted sensitivities were:

$$S_{\text{CO InfraTec}} = 0.08139, \qquad S_{\text{CO a-DBR}} = 0.01989$$

yielding an enhancement ratio of:

$$E_{\text{CO, matched FWHM}} = 0.24441$$

The reduced relative sensitivity of the a-DBR emitter in the unmatched-bandwidth case reflects the significantly broader passband of the commercial NDIR filter, which integrates over a larger spectral range. This result highlights the fundamental trade-off between broadband signal throughput and narrowband

spectral selectivity. While broadband filters may yield higher absolute sensitivity when bandwidth is unconstrained, the a-DBR emitter enables precise spectral isolation of the target absorption feature, which is critical for multi-gas discrimination and independent detection of spectrally similar species.

S13. CO_2 Emitter Comparison to Commercial NDIR Gas Sensor

This section evaluates the sensitivity of the spectrally selective CO_2 a-DBR emitter relative to another commercial InfraTec NDIR filter for CO_2 gas detection.[11] The CO_2 a-DBR emitter exhibits a single emission resonance centered at 2351 cm^{-1} with an FWHM of 36.91 cm^{-1} and a peak emissivity of 0.528. For comparison, the commercial InfraTec CO_2 NDIR filter is centered near 2347 cm^{-1} with a FWHM of 50 cm^{-1}, and a peak transmission of 0.805.

To enable a more direct comparison, consistent with the approach used in Section S.12, two sensitivity analyses were performed. In the first case, the InfraTec filter response was recalculated using a matched FWHM of 36.91 cm^{-1}, equivalent to the linewidth of the a-DBR emitter, consistent with the approach used for the C_3H_8 and CO emitters. Under matched-bandwidth conditions, the integrated sensitivities were found to be: $S_{\mathrm{CO_2\ InfraTec,\ matched\ FWHM}} = 0.11677, \qquad S_{\mathrm{CO_2\ a\text{-}DBR,\ matched\ FWHM}} = 0.07542.$

The resulting enhancement ratio is:

$$E_{\mathrm{CO_2,matched\ FWHM}} = 0.646,$$

corresponding to a 35.4% reduction in sensitivity relative to the commercial InfraTec CO_2 NDIR filter. This reduced enhancement arises because the peak emissivity of the CO_2 a-DBR emitter is substantially lower than the peak transmission of the InfraTec filter. Given that the bandwidths are matched and the spectral centers are closely aligned, the observed sensitivity is primarily governed by this difference in peak intensity.

In the second case, sensitivities were calculated using the unmatched bandwidths of each component to reflect their typical operating conditions. Using the InfraTec filter FWHM of 50 cm^{-1} and the a-DBR emitter FWHM of 36.91 cm^{-1}, the extracted sensitivities were:

$$S_{\mathrm{CO_2\ InfraTec}} = 0.14712, \qquad S_{\mathrm{CO_2\ a\text{-}DBR}} = 0.07542,$$

yielding an enhancement ratio of:

$$E_{\mathrm{CO_2}} = 0.513.$$

The reduced sensitivity under unmatched-bandwidth conditions reflects the broader bandwidth of the commercial filter. Again, the primary objective of the CO and CO_2 a-DBR emitters is enhanced spectral selectivity rather than maximum sensitivity, as discussed in the main text, where their ability to discriminate between closely spaced CO and CO_2 absorption features is demonstrated.

References

(1) Joannopoulos, J. D.; Johnson, S. G.; Winn, J. N.; Meade, R. D. *Photonic Crystals: Molding the Flow of Light - Second Edition*; Princeton University Press, 2011. https://doi.org/10.1515/9781400828241.

(2) He, M.; Nolen, J. R.; Nordlander, J.; Cleri, A.; McIlwaine, N. S.; Tang, Y.; Lu, G.; Folland, T. G.; Landman, B. A.; Maria, J.-P.; Caldwell, J. D. Deterministic Inverse Design of Tamm Plasmon Thermal Emitters with Multi-Resonant Control. *Nat. Mater.* **2021**, *20* (12), 1663–1669. https://doi.org/10.1038/s41563-021-01094-0.

(3) mingze321. Mingze321/Tamm, 2021. https://github.com/mingze321/Tamm (accessed 2025-10-23).

(4) *Linkam THMS600 - Temperature Control Stage for Microscopy and Spectroscopy*. Linkam Scientific. https://www.linkam.co.uk/thms600 (accessed 2025-10-23).

(5) *Electroformed Parabolic Reflectors*. https://www.optiforms.com/electroformed-components/parabolic-reflectors/ (accessed 2025-10-23).

(6) PIKE-Technologies_Stainless-Steel-Short-Path-Gas-Cells.Pdf. https://www.piketech.com/wp-content/uploads/PDS/transmission/PIKE-Technologies_Stainless-Steel-Short-Path-Gas-Cells.pdf (accessed 2025-10-23).

(7) *Alicat Mass Flow Controller with Display, 0 - 200 SCCM from Cole-Parmer*. https://www.coleparmer.com/i/alicat-mass-flow-controller-with-display-0-200-sccm/1530902?PubID=UX&persist=true&ip=no&gad_source=1&gad_campaignid=21168216896&gbraid=0AAAAAD-n1iXfA7D34vlhqAd6H1yLgJlXn&gclid=CjwKCAjwpOfHBhAxEiwAm1SwEhJG4K7x0MfHt-KoKNBrwos-GO67HQvQCzqhLueQXDDFuk4gWhg0nBoCAdAQAvD_BwE (accessed 2025-10-23).

(8) *Thorlabs - MPD169-P01 Ø1*. https://www.thorlabs.com (accessed 2025-10-23).

(9) *RM9-Pyro 100 nW to 100 mW Pyroelectric Sensor Radiometer*. https://www.ophiropt.com/en/f/rm9-pyro-radiometer (accessed 2025-10-23).

(10) Standardfilter_Katalog+homepage_Layout.Xlsx. https://media.infratec.eu/infratec-b-filter-homepage-layout.pdf?mp_enc=bXBfZGlyPTY1MTY3Jm1wX2ZpbGU9NDc3OTM0MjM= (accessed 2025-10-23).

(11) *Get IR filters and IR windows from InfraTec*. https://www.infratec-infrared.com/sensor-division/ir-filters/ (accessed 2025-10-06).
